# University students’ preferences of learning modes post COVID-19-associated lockdowns: In-person, online, and blended

**DOI:** 10.1371/journal.pone.0296670

**Published:** 2024-07-19

**Authors:** Kosha J. Mehta, Javier Aula-Blasco, Julia Mantaj

**Affiliations:** 1 Centre for Education, Faculty of Life Sciences & Medicine, King’s College London, London, United Kingdom; 2 Institute for Education, Teaching and Leadership, University of Edinburgh, Edinburgh, United Kingdom; 3 School of Cancer and Pharmaceutical Sciences, Faculty of Life Sciences & Medicine, King’s College London, London, United Kingdom; 4 Faculty of Science & Engineering, School of Life Sciences, Anglia Ruskin University, Cambridge, United Kingdom; UCL: University College London, UNITED KINGDOM

## Abstract

Online teaching accelerated during COVID-19-associated lockdowns. At that time, it was assumed that university students wanted to revert to in-person sessions at the earliest opportunity. However, when in-person sessions were re-introduced, student attendance was not as high as expected. Therefore, we examined students’ preferences of learning modes. Students (n = 968) from different UK universities, degree cohorts, study levels and biological sexes were given four learning-mode options: Face-to-face sessions for lectures and tutorials (in-person), Live online sessions for lectures and tutorials (Online-synchronous), Pre-recorded lectures and live online tutorials (Online-mixed-asynchronous-synchronous), and Pre-recorded lectures and face-to-face tutorials (Blended: in-person and online-asynchronous). Students ranked these options as per their preference via an online anonymous survey. Data were analysed using IBM SPSS Statistics 28. Results showed that the most frequently selected 1^st^ and last choices were In-person and Online-synchronous modes, respectively. For the majority, above choices were the same across study levels and biological sex, but across degree cohorts, the 1^st^ choice was either In-person or Blended. Proportion of students selecting In-person mode as their 1^st^ choice (52.2%) was almost equal to the combined proportions of those selecting other learning modes as 1^st^ choices (47.5%). Amongst degree cohorts, In-person mode was least preferred by Language Education students and most preferred by Bioscience and Sports & Exercise Science students. The latter cohort also preferred Online-synchronous mode more than other degree cohorts. Blended mode was preferred more by Language Education, Computer Science and Psychology students but preferred less by Sports & Exercise Science and Pharmacy students, compared to other degree cohorts. Ordinal regression revealed that Sports & Exercise Science students preferred Online-mixed-asynchronous-synchronous mode less than Language Education students. Undergraduates preferred In-person mode more and Online-mixed- asynchronous-synchronous mode less than postgraduates. Preference differences between biological sexes were insignificant. Thus, we identified students’ preferences of learning modes and propose that not biological sex, but discipline and study level can predict/influence preferences.

## Introduction

Until about three decades ago, most university teaching was conducted in-person. With the advent and establishment of digital technology in education, universities began to offer online teaching activities as part of regular programs. The use of online teaching was greatly accelerated worldwide following the onset of the COVID-19 pandemic. In that period, education providers extensively utilised digital platforms to provide education online [1, 2] resulting in an unprecedented level of experience of online learning amongst students.

During the peak period of the pandemic, several elite institutions in the US had “substantially discounted tuition for their fully online experience” (Harvard Business Review-29/9/2020). Around this time, in the UK, a petition was registered to parliament with 270,659 signatures. It stated that the quality of online education was not equal to in-person teaching and as such, university students should not have to pay full tuition fees for the academic year 2020-2021without experiencing university life. This topic was subsequently debated in the UK parliament on 16^th^ November 2020 [3] with substantial mainstream media coverage. This was followed by BBC News article dated 9/9/2021 entitled “Universities have been urged to provide in-person teaching when students return this term”. The article included statements from the UK Education Secretary who expressed the need for in-person teaching and suggested that online education should only be used where technology would provide a “genuine benefit”.

These events and media coverage led to the assumption that fully online teaching was a temporary fix and that university students wanted to revert to in-person teaching at the earliest opportunity. Accordingly, following the launch of the COVID-19 vaccination program and easing of restrictions, around 1.5 years after the declaration of the pandemic, universities started to return to in-person teaching. The UK petition had implied that students valued in-person teaching more than online teaching and, therefore, it was expected that when in-person teaching recommenced, attendance at these sessions would be high. However, the consensus among academics has been that student attendance was not as high as expected in lectures and tutorials. Moreover, the attendance seemed to dwindle as the year progressed. The Times Higher Education (9/6/2022) confirmed this and reported a drop in class attendance globally post COVID-19 [4, 5].

This was an unexpected scenario which exposed an apparent paradox between what students said they wanted and what they did in practice. This poses baffling but important questions around how exactly students want to learn and what is their preferred mode of learning. Furthermore, the post-pandemic era arguably offers the best possible opportunity to investigate preferences of learning mode because, for the first time in history, all the student body will have experience of both online and in-person teaching. Also, students’ choice of learning mode can influence engagement, retention, and attainment [6, 7]. Thus, it is important to know students’ choices clearly so that the data can be used by academic institutions to make informed decisions when designing learning modes for future teaching sessions and courses.

Therefore, here, we examined students’ preferences for different learning modes and investigated whether these varied by student characteristics such as discipline, study level, and biological sex. Accordingly, the objectives of this study were i) to examine students’ preferred learning modes for lectures and tutorials and determine the most frequently selected first and last choices, ii) to ascertain whether students’ preferences varied between discipline (degree cohorts), study levels and biological sexes, and iii) to assess whether degree cohort, study level or biological sex could predict the ranking/preferences for these learning modes.

### Review of literature on students’ preferred mode of learning

There have been several studies that examined students’ preferences of learning modes prior to and during the pandemic. For example, in a pre-pandemic study, a small number of medical students at the University of Ulm (Germany) preferred face-to-face electrocardiogram course over the e-learning course despite the similarity between the two courses. Generally, the students felt that face-to-face sessions offered several positives such as direct interaction with subjects/academics, provision of fixed time, and the requirement to be physically present that led to better commitment to the course [8]. Similarly, in an Australian study, about 70% of physiology students showed preference for face-to-face teaching-learning [9]. On the other hand, engineering students in California State University indicated the cons of learning by digital means. More than half of the students indicated that online education was fraught with issues like technical problems, challenges in teaching and learning, concerns of security and privacy, sub-optimal engagement in class and online fatigue, specifically after attending multiple sessions [10]. Along similar lines, during the COVID-19 pandemic, although the university students in Saudi Arabia highlighted the convenience offered by online education, they also felt that the quality of education and the amount of knowledge gained had decreased. Engagement was negatively affected and lack of motivation to study prevailed amongst students [11]. Hollister et al. (2022) reviewed a study by Means and Neisler (2020) that was conducted around the time of the pandemic. They stated that from about a thousand undergraduates who began courses in-person and ended online, approximately half of them were very satisfied with their course prior to the pandemic and only one-fifth were very satisfied after transition to online learning. The most likely reasons for this being difficulty in maintaining interest and motivation, and the lack of collaboration with peers [7].

These studies collectively bring forth students’ preference for in-person learning. Face to-face sessions are important because personal interaction with the lecturer is undeniably an extremely important part of the learning process. The lecturer can direct and motivate. Therefore, this should take precedence when designing courses, as also opined by others [8].

Notably, there are studies that reported students’ inclination for the blended mode. For example, in an Australian university, about 30% of undergraduate nursing students studying anatomy and physiology suggested that laboratory classes were most impactful, but about 45% students agreed that recorded lectures were also useful [12]. This inclination for a blended approach has been supported by another study which showed that although students from the Lucerne University of Applied Sciences and Arts (Switzerland) preferred to see return of face-to-face sessions, about 42.5% of students alluded to the idea that in addition to the above, higher levels of distance learning should be targeted for the post-pandemic period [13].

It is important to note that due to the pandemic-associated lockdowns, students faced several challenges including mental health issues. In-person social interaction had greatly reduced, and this aggravated the symptoms of depression and feelings of loneliness amongst students [14–16]. Bearing this, indeed, the preference can change with time and circumstance for an individual student.

## Methods

### Participants

Data were collected from 968 students at two Russell Group (research intensive) universities in the UK. Participating students were from two study levels (undergraduate and postgraduate) and hailed from six different degree cohorts, namely Bioscience, Psychology, Pharmacy, Computer Science, Sports & Exercise Science, and Language Education. Ethical approval was obtained by the corresponding author/principal investigator from the Research Ethics Office of King’s College London, UK; LRS-19/20-19743). Approval was also obtained from the other participating university via email by the academic collecting the data at that university.

### Data collection

Prospective participants were sent a participant information sheet at least 24 hours prior to the teaching session in which data were to be collected. These data were collected as a part of a larger survey during synchronous teaching, either in-person or online. Data were collected anonymously in the following manner. On the day of data collection, before the teaching commenced, the participants were asked to self-design a screen name (code name) for themselves (to maintain student anonymity) and use this to login and provide their responses. This was done on a digital platform; PollEV or Qualtrics software depending on availability to individual lecturers. Prior to the analysis of data, even the screen names were removed, and data were thus analysed anonymously. At the end of the teaching session, students were asked to rank the following learning modes in order of preference: Face-to-face sessions for lectures and tutorials (in-person mode), Live online sessions for lectures and tutorials (Online-synchronous mode), Pre-recorded lectures and live online tutorials [Online-mixed-asynchronous (async)-synchronous (sync)], and Pre-recorded lectures and face-to-face tutorials (Blended mode involving in-person and online-asynchronous modes).

The participant information sheet that was sent to students prior to the day of data collection included information on how the data would be collected and statements like ‘Participation in the survey or the topic test is not compulsory’ and ‘Your participation implies your consent for analysis of the data, its publication in journals and/or presentation at conferences’. The participant information sheet also included a provision for withdrawal from the study. For example, if a student participated, but later decided to withdraw, then within 72 hours of taking part in the survey, they could reveal their unique anonymous code that they created previously, and their responses would be excluded from the study. This was reiterated verbally to the students at the start of the sessions prior to data collection. Furthermore, the 1^st^ question on the survey asked students again whether they wished to participate. The submission of their responses to survey questions implied their consent to participate in the study (unless they decided to withdraw later). Thus, informed consent was obtained digitally from all participants in this way.

Data were collected over a period of two years (from November 2020 to December 2022). This covered the phases of pandemic peaks (representing surges in COVID-19 cases and deaths), pandemic-associated lockdowns, and the period after introduction of the COVID-19 vaccination programme.

### Statistical analyses

All statistical analyses were conducted using IBM SPSS version 28. Differences in ranking/preference between the four learning modes were examined via Friedman test. Post-hoc analysis was done via Wilcoxon signed-ranks test with Bonferroni correction. Differences in distribution of ranking/preferences of a learning mode between students of different degree cohorts, study levels or biological sex were examined using Chi-squared (*X*^2^) test [17]. Ordinal logistic regression was used to assess whether degree cohort, study level or biological sex could predict the preference order/ranking of the four learning modes [18]. We used ordinal logistic regression because the outcome variable i.e., ranking of the learning mode is ordinal (categories have an order). We regressed each learning mode with one predictor variable (degree cohort, study level or biological sex) at a time. In the context of model fit, our null hypothesis was that there was no significant difference between baseline models to final models. Odds ratio (ExpB) was expressed with 95% Wald confidence interval (CI). Test results were considered significant when p<0.05.

## Results

### Sample characterisation

**Fig 1:**
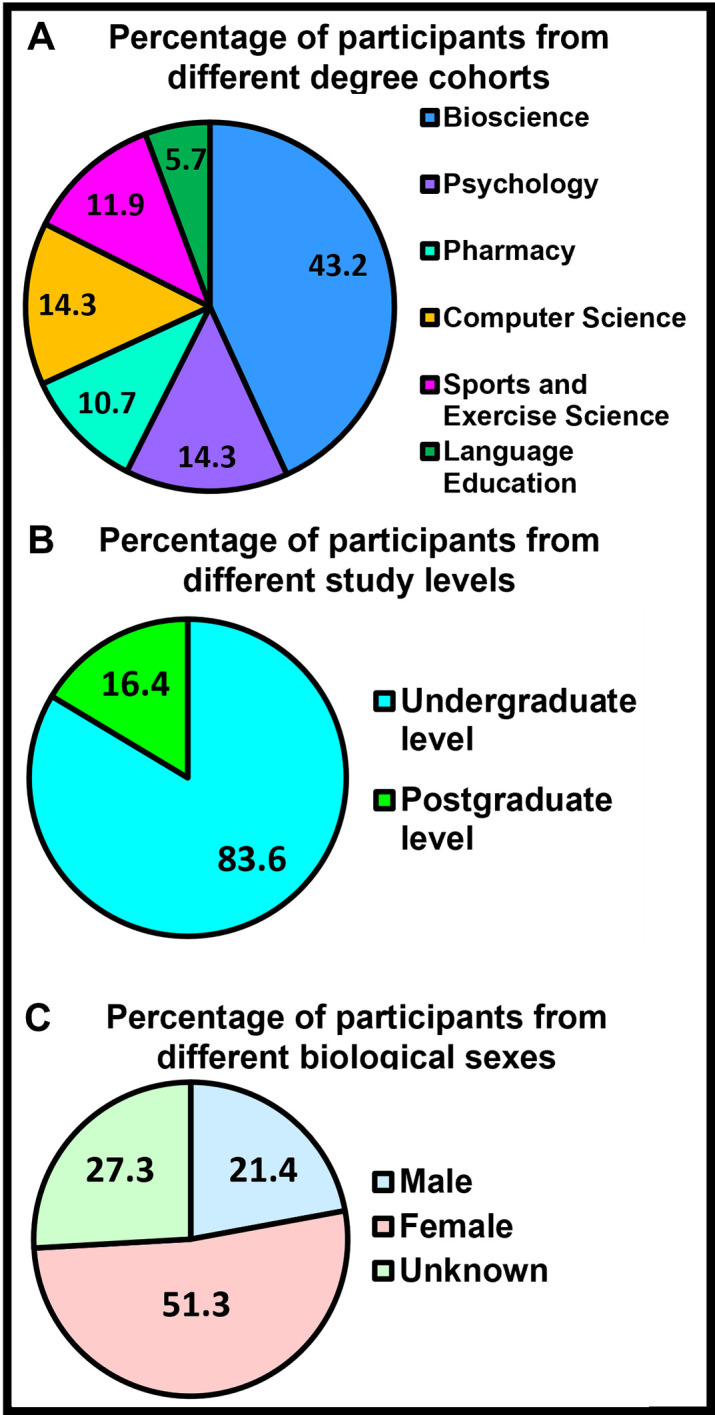
Percentage of participants from different degree cohorts, study levels and biological sexes. A: The figure depicts the proportion of participating students from six degree cohorts. The highest proportion of participants were from Bioscience whereas the lowest proportion of participants were from Language Education. The proportions of students from other degree cohorts were similar. B: The figure depicts the proportion of participating students from two study levels i.e., undergraduates and postgraduates. A vast majority of participating students were undergraduates. C: The figure depicts the proportion of participating students from the two known biological sexes. While there were more female participants than male participants, the biological sex of a proportion of participants remained unknown because these participants did not reveal their biological sex.

Fig 1 depicts percentage of participants from six different degree cohorts, two different study levels and biological sexes. Essentially, most participants were from Biosciences (Fig 1A), undergraduate level (Fig 1B) and female (Fig 1C). The least number of participants were from Language Education (Fig 1A), postgraduate level (Fig 1B), and male (Fig 1C). It is noteworthy that around 50% of participants did not reveal their sex.

### Overall student preferences: In-person mode preferred by majority

**Fig 2:**
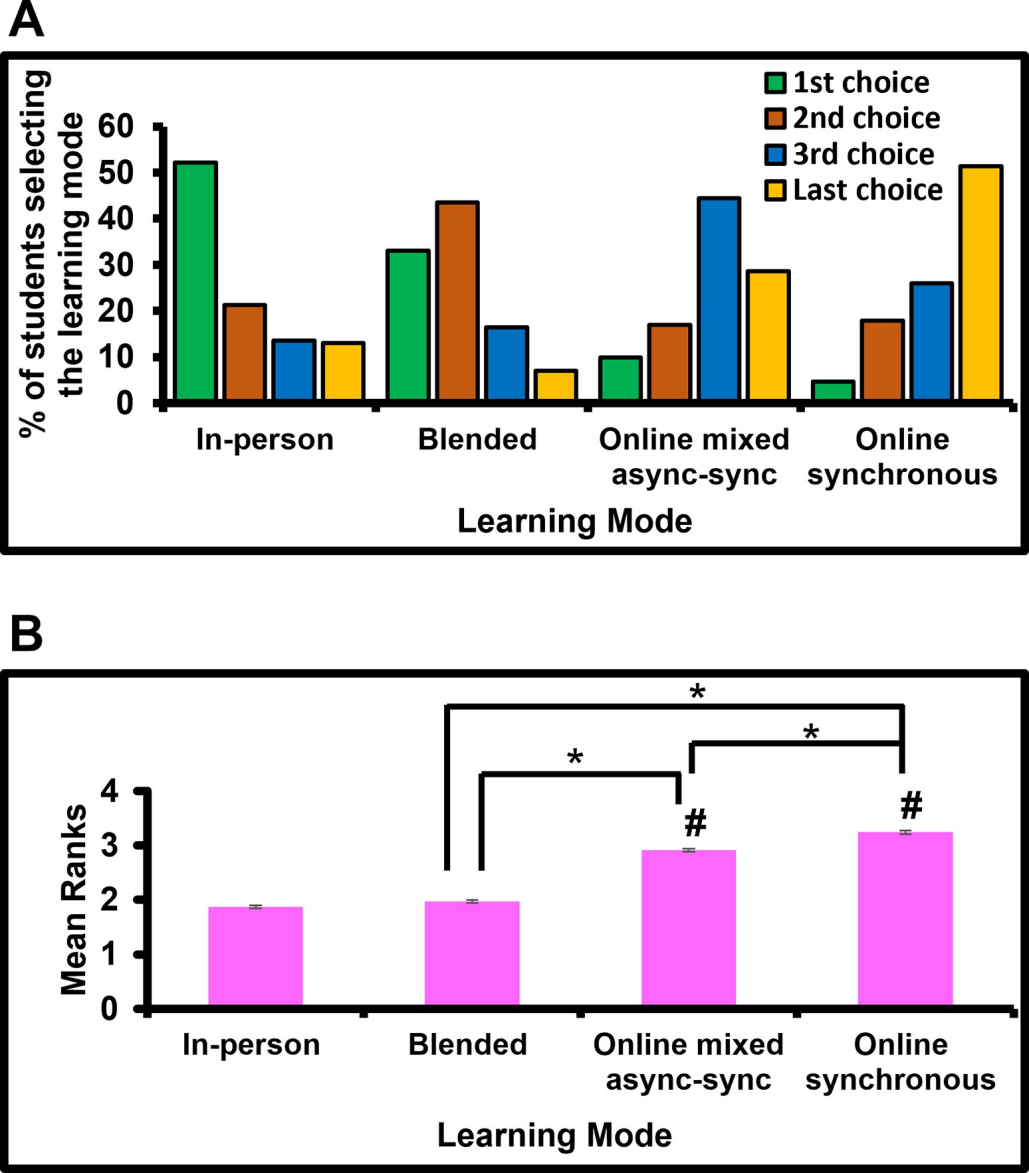
Most frequently selected choices of learning modes overall. A: The figure shows the percentage of students selecting each learning mode as 1^st^, 2^nd^, 3^rd,^ and last choices for lectures and tutorials. In-person mode was the most frequently selected 1^st^ choice while Online-synchronous mode was the most frequently selected last choice. B: The figure shows mean ranks of each learning mode with standard error of mean. *p<0.001 reflects statistical differences between groups, as indicated on figure and ^#^p<0.001 reflects differences with the In-person mode.

Overall, 52.2% of students selected In-person as their 1^st^ choice of learning mode, and collectively, 47.5% students selected the other learning modes as their 1^st^ choices. Fig 2A details the percentage of students selecting each learning mode as their 1^st^, 2^nd^, 3^rd,^ and last choices. Essentially, the most frequently selected 1^st^ choice was the In-person mode. The most frequently selected 2^nd^ and 3^rd^ choices were Blended and Online-mixed-async-sync modes, respectively. The most frequently selected last choice was the Online-synchronous mode (Fig 2A), as selected by 51.4% students.

Fig 2A indicated inequalities in preferences i.e., inequalities in the ranking of the four learning modes by students. So, we conducted statistical analyses to reconfirm the above and examine whether there were any significant differences in ranking/preference of the four learning modes. Friedman’s test revealed a statistically significant difference in preferences for the four learning modes [*χ*^*2*^ (3) = 810.93, p<0.001]. Post-hoc analysis with Wilcoxon signed-ranks test and Bonferroni correction (α<0.008) indicated that the preferences/ranking of In-person mode significantly differed from the preferences/ranking of the other learning modes (p<0.001), except Blended mode (Z = -1.79, p = 0.73). Also, the preferences/ranking of Online-synchronous mode significantly differed from that of Blended mode (Z = -19.5, p<0.001) & Online-mixed-async-sync mode (Z = -6.5, p<0.001). Likewise, the preferences/ranking of Blended mode differed from that of Online-mixed-async-sync mode (Z = -18.1, p<0.001).

The mean rank for each learning mode is shown in Fig 2B where a lower rank (i.e., 1) indicates a stronger preference for the mode than a higher rank (i.e.,4). This analysis re-confirmed that the In-person mode was ranked as the more preferred in contrast to all others and the least preferred choice was Online-synchronous (p<0.001).

### Students’ preferences of learning modes across degree cohorts (discipline)

The above analyses determined the most preferred and least preferred choices for the entire student population in this study. However, we also aimed to examine if preferences differed by student characteristic.

Considering the characteristic of discipline, we determined the most frequently selected first and last choices within different degree cohorts i.e., disciplines of study, as shown in Table 1.

**Table 1:** First and last choices of learning modes chosen by majority of students in the different degree cohorts.

Degree cohort	Most frequently selected 1^st^ choice of learning mode & percent of students making this selection	Most frequently selected last choice of learning mode & percent of students making this selection
Bioscience	In-person (57.2%)	Online-synchronous (49.8%)
Pharmacy	In-person (49.0%)	Online-synchronous (59.6%)
Sports & Exercise Science	In-person (67.8%)	Online-mixed-async-sync (42.6%)
Psychology	• In-person (42.0%) • Blended (42.0%)	Online-synchronous (60.1%)
Computer Science	Blended (42.8%)	Online-synchronous (55.8%)
Language Education	Blended (49.1%)	Online-synchronous (52.7%)

**Fig 3:**
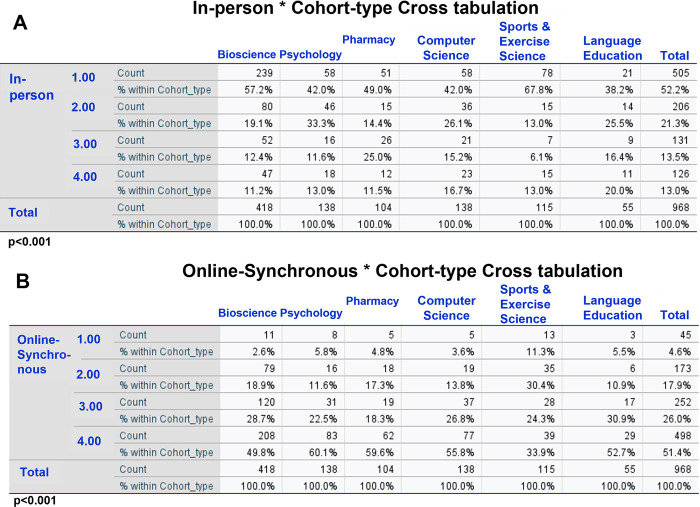
Differences in ranking of learning modes across degree cohorts. The figure shows the results of Chi-squared tests and indicates the differences in the ranking of In-person mode (A) and Online-synchronous mode (B), and thereby the differences in proportion of students selecting these modes across degree cohorts.

Chi-squared test showed significant differences between degree cohorts in the distribution of ranking (preference) of the In-person mode [*X*^2^ (15) = 54.1, p<0.001] (Fig 3A). Here, a substantial proportion of students from Sports & Exercise Science (68%) and Bioscience (57%) selected In-person mode as 1^st^ choice. This indicated that the In-person mode was preferred more by these students compared to students on other degree cohorts, including Language Education students of which only 38% selected this mode as their 1^st^ choice (Fig 3A). Essentially, across all degree cohorts, students of Sports & Exercise Science and Bioscience showed highest/more preference for In-person mode, while Language Education students least preferred the In-person mode.

Evaluating the most frequently selected last choice i.e., Online-synchronous mode, Chi-squared test showed significant differences between degree cohorts in the distribution of ranking (preference) of this mode [*X*^2^ (15) = 47.5, p<0.001] (Fig 3B). Data showed that a third of Sports & Exercise Science students, but about half or more than half (50–60%) students on other degree cohorts selected this mode as their last choice (Fig 3B). Collectively, data indicated that while Online-synchronous mode was the least preferred mode across all degree cohorts, Sports & Exercise Science students preferred this mode more than students on other degree cohorts.

We additionally examined the responses of students to Online-mixed-async-sync mode. Chi-squared test identified significant differences between degree cohorts in the preference of this mode [*X*^2^ (15) = 46.2, p<0.001]. This mode was most preferred i.e., selected as 1^st^ choice by Pharmacy students (17.3%) and least preferred as 1^st^ choice by students of Sports & Exercise Science (8.7%) and Language Education (7.3%).

### Students’ preferences of learning modes across study levels

**Fig 4:**
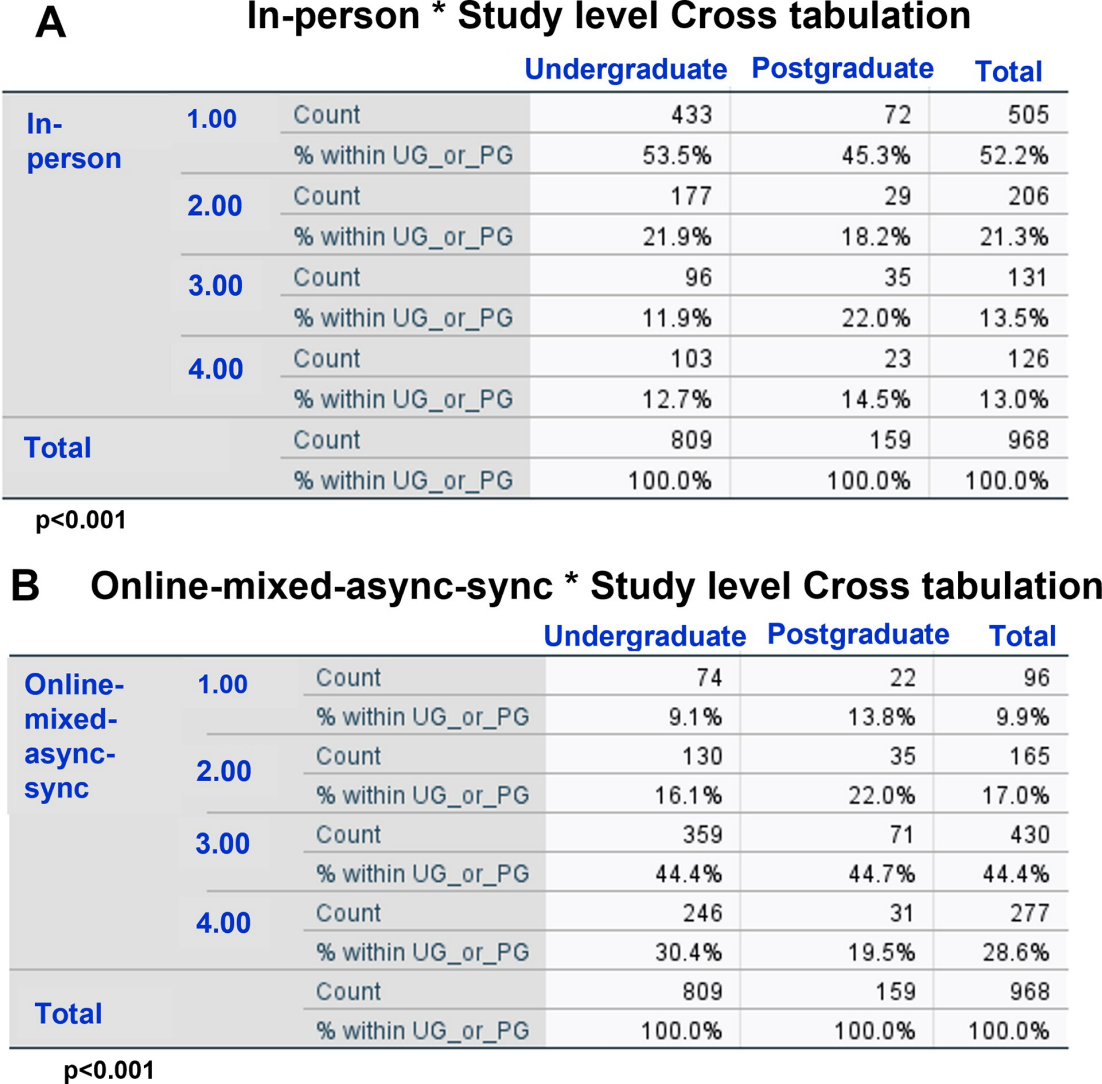
Differences in ranking of learning modes across study levels. The figure shows the results of Chi-squared tests and indicates the differences in the ranking of In-person mode (A) and Online-mixed-async-sync mode (B), and thereby the differences in proportion of students selecting these modes across study levels.

At study level, the choices seemed to be the same as that of the overall student population; In person and Online-synchronous being the most frequently selected 1^st^ and last choices, respectively. Chi-squared tests showed significant differences between study levels in the distribution of rankings of the In-person mode [*X*^2^ (3) = 12.9, p = 0.005] (Fig 4A). A greater proportion of undergraduates reported In-person as their first choice (53.5%) compared to postgraduates (45.3%). Essentially, the In-person mode was preferred more by undergraduates compared to postgraduates.

There was no difference in distribution of Online-synchronous across study levels (p = 0.3), although a greater proportion of postgraduates reported Online-synchronous as their least preferred choice (undergraduates: 50.3%, postgraduates: 57.2%). The preference for Online-mixed-async-sync mode significantly differed between study levels [*X*^2^ (3) = 11.2, p = 0.01] (Fig 4B) with more postgraduates than undergraduates preferring this mode, as indicated by higher proportion of postgraduates selecting this mode as 1^st^ choice and a lower proportion of these students selecting this mode as their last choice, compared to undergraduates (Fig 4B).

### Students’ preferences of learning modes across biological sex

Considering the characteristic of biological sex, frequency data showed that the overall preferences remained the same (i.e., In-person was most preferred and Online-synchronous was least preferred for any category of sex). In context, for each one of the four learning modes, Chi-squared tests showed no significant differences between the 3 groups of males, females, and unknown biological sex (i.e., the group in which biological sex remained unknown). Data were re-analysed using only two groups wherein the biological sex was known, i.e., utilising male and female groups only. The results remained the same with no statistical differences between these groups. However, the percentage of students showing these preferences differed slightly. For example, In-person mode was preferred by a greater proportion of males (56.5%) compared to females (48.3%), with those of unknown gender showing similar preference (56.1%) to males. The differences in the ranking of the Online-synchronous mode between sexes were smaller (52.7% males, 50.7% females and 51.9% students with unknown biological sex selected this mode as their last choice).

### Analyses of the 2^nd^ best overall choice: The Blended mode

In addition to evaluating the most frequently selected 1^st^ choice (In-person) and last choice (Online-synchronous), we wanted to examine the patterns and proportions of students selecting the 2^nd^ best choice reported overall i.e., the Blended learning mode (Fig 2A). As such, Jisc news dated 7/09/2022 reported that majority of university students wanted blended learning, as concluded from a survey. Therefore, we examined the preference for Blended mode in our study, and further assessed whether there were differences in students’ preference for Blended mode based on their discipline, level of study or biological sex.

**Fig 5:**
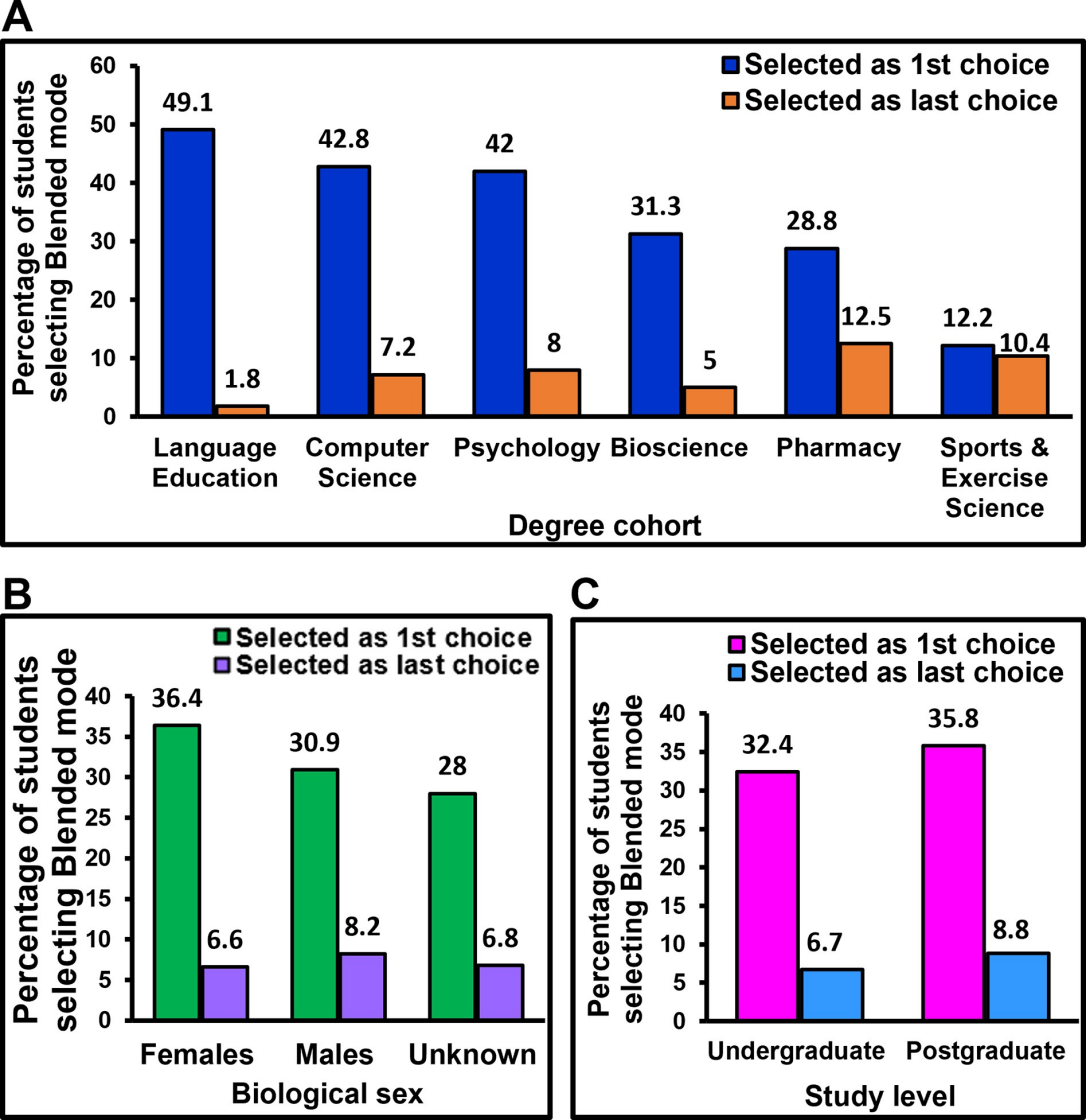
Percentage of students selecting Blended mode for lectures and tutorials. The figure depicts the percentage and thereby the proportion of students that selected Blended mode as their 1^st^ and last choices. A: Amongst all degree cohorts, the highest proportion of students selecting this mode as 1^st^ choice was from Language Education. Students of Sports & Exercise Science least selected this mode as the 1^st^ choice compared to the other degree cohorts. B: About a third of both male and female students selected this mode as their 1^st^ choice. This is bearing that the biological sex of a proportion of participants remained unknown. C: About a third of both undergraduates and postgraduates selected this mode as the 1^st^ choice.

Fig 5 shows the percentage of students that selected Blended mode for lectures and tutorials within each degree cohort, study level and biological sex. Blended mode was selected as their 1^st^ choice by almost half of Language Education students, more than one third of Computer Science and Psychology students and, about a third of Bioscience and Pharmacy students (Fig 5A). Amongst all degree cohorts, Sports & Exercise Science students least preferred the Blended mode as their 1^st^ choice (12.2% students) (Fig 5A). Chi-squared test confirmed that preferences for Blended mode significantly differed between different degree cohorts [*X*^2^ (15) = 66.7, p<0.001], with more students of Language Education, Computer Science and Psychology preferring Blended learning as their 1^st^ choice compared to students of other degree cohorts. Essentially, Blended mode was preferred more by Language Education, Computer Science and Psychology students but preferred less by Sports & Exercise Science and Pharmacy students, compared to students on other degree cohorts.

Upon considering biological sex as a parameter, about a third of both male and female populations selected this mode as the 1^st^ choice (Fig 5B). The percentage of students that selected this mode as the last choice was similar in both populations (Fig 5B). Chi-squared tests revealed no significant differences in preferences of this mode between groups of biological sex, whether known or unknown.

Evaluating preferences in the context of study level, about a third of both undergraduates and postgraduates selected Blended mode as the 1^st^ choice, with no major difference in proportion of students selecting this mode as the last choice (Fig 5C). Chi-squared tests confirmed that there were no significant differences between study levels in the preference of this mode (p = 0.2).

### Predicting preferred learning mode by degree cohort, study level and biological sex

Next, we aimed to examine whether degree cohort, study level or biological sex could predict the preference of the four learning modes, i.e., predict the way in students ranked a certain learning mode.

### Degree cohort

For degree cohort, in case of In-person and Blended modes, our null hypothesis (stated in Methods) was retained. This is because for both these learning modes, although the final model showed significant difference from the intercept only model, [*χ*^*2*^ (5) = 25.9, p<0.001] and *χ*^*2*^ (5) = 47.8, p<0.001, respectively], the Goodness-of-fit Pearson values and Test of parallel lines Chi-Square showed significant results (p<0.05). This indicated a poor fit of model. Therefore, the models were not considered suitable for prediction of ranking of these learning modes by degree cohort.

In contrast, the models generated for the modes Online-synchronous and Online-mixed-async-sync rejected the null hypothesis/models and were found fit for prediction of the ranking of these learning modes [*χ*^*2*^ (5) = 30.87, p<0.001, and *χ*^*2*^ (5) = 33.7, p<0.001, respectively] with both Goodness-of-fit Pearson values and Test of parallel lines Chi-Square values greater than 0.05, indicating a good model fit.

Accordingly, for Online-synchronous mode, data showed that compared to students of Language Education, for students of Sports & Exercise Science lower cumulative scores were more likely [Estimate (β): -0.97, Exp(B) (odds ratio): 0.37 (CI: 0.2–0.60), p = 0.002]. This indicated that compared to students of Language Education, students in Sports & Exercise Science had an increased likelihood of selecting a lower rank, and there were lower odds of selecting higher ranks for this learning mode. Since in this study, a lower rank reflected higher preference (1^st^ choice being ranked as 1 and last choice being ranked as 4), this data implied that compared to Language Education students, Sports & Exercise Science students showed a higher preference (reflected as increased likelihood of selecting a lower rank) for Online-synchronous mode (as also reflected in Fig 3B). Other degree cohorts did not significantly predict such relationships with this learning mode.

For Online-mixed-async-sync mode, data showed that compared to students of Language Education, for students of Sports & Exercise Science, higher cumulative scores were more likely [Estimate (β): 0.72, Exp(B) (odds ratio): 2.05 (CI: 1.1–3.7), p = 0.01]. This indicated that compared to students of Language Education, students in Sports & Exercise Science had an increased likelihood of selecting higher ranks for this learning mode, and there were higher odds of selecting higher ranks for this learning mode. Since in this study, a higher rank reflected lower preference (1^st^ choice being ranked as 1 and last choice being ranked as 4), the data implied that compared to Language Education students, Sports & Exercise Science students showed a lesser preference (reflected as increased likelihood of selecting a higher rank) for this learning mode. Other degree cohorts did not significantly predict such relationships with this learning mode.

### Study level

In case of study level, our null hypothesis was retained for In-person, Blended, and Online-synchronous modes. The models were not considered suitable for prediction of the ranking of these learning modes. For In-person mode, although the final model showed significant difference from the intercept only model, [*χ*^*2*^ (1) = 5.3, p = 0.02], the Goodness-of-fit Pearson value and Test of parallel lines Chi-Square showed significant results (p<0.05). This indicated a poor fit of model. For Online-synchronous mode [*χ*^*2*^ (1) = 2.0, p = 0.1] and Blended mode [χ^2^ (1) = 0.9, p = 0.3], the final models were not significantly different from the intercept only model.

In contrast, for Online-mixed-async-sync mode, the model was found fit for prediction of ranking of this learning mode by study level [*χ*^*2*^ (1) = 11.16, p<0.001) with both Goodness-of-fit Pearson values and Test of parallel lines Chi-Square greater than 0.05. Data showed that compared to postgraduates (used as reference category), for undergraduates, higher cumulative scores were more likely [Estimate (β): 0.53, Exp(B) (odds ratio): 1.7 (CI: 1.2–2.3), p< 0.001]. This indicated that compared to postgraduates, undergraduates had a higher likelihood of selecting higher ranks for this learning mode, and there were higher odds of selecting higher ranks for this learning mode. Since in this study, a higher rank reflected lower preference (1^st^ choice being ranked as 1 and last choice being ranked as 4), the data implied that compared to postgraduates, undergraduates showed a lesser preference (reflected as increased likelihood of selecting a higher rank) for this learning mode.

### Biological sex

Since the biological sex for a proportion of students was not known, for prediction studies, we selectively analysed data in cases where the biological sex was known i.e., either male or female, to enable the examination whether biological sex could predict the ranking/order of the learning modes. Analysis of model fitting information revealed that our null hypothesis was retained for all learning modes because there were no significant differences between baseline/intercept models to final models. Therefore, the models were not considered suitable for prediction of the ranking of the learning modes.

## Discussion

Students seem to value in-person learning more than online learning but when the in-person mode was re-introduced following the ease of COVID-19-related restrictions, students’ attendance was not as high as expected. This posed important questions around how students want to learn. Therefore, this study aimed to examine students’ preferences/choices of learning modes.

In this broad study, instead of recruiting students from a single institution and from the same degree cohort, we recruited students from different universities, six different degree cohorts, two different study levels, and the data were collected over a long period of 2 years. Here, students were presented with four learning modes which included lectures and tutorials as session types, and in-person, blended and fully online as teaching-learning modes. Students were asked to rank the four learning modes in the order of their preference. We aimed to determine their most and least selected 1^st^ and last choices and examine whether their choices (and the ranking order of the four modes) differed between degree cohorts, study levels and biological sex.

### The expected: Overall, students prefer In-person mode

Majority of students selected the In-person mode as their 1^st^ choice and Online-synchronous mode as their last choice (Fig 2A and 2B). These data support other studies which showed that undergraduate students at British universities preferred in-person learning to blended learning when COVID-19 is not considered [19, 20]. Along similar lines, a pre-pandemic study showed that undergraduate students at an Australian university studying Psychology preferred completing the assigned activities in-person rather than online. Students felt that in-person class discussions (rather than online) fostered more engagement and therefore preferred in-person class discussions [21]. In yet another study conducted during the COVID-19 pandemic, about 40% of participating undergraduate nursing students at an Australian university preferred face-to-face sessions for studying human anatomy and physiology [22]. Simultaneously, studies have shown the student-perceived downside of using digital technology [23]. Online learning has been perceived as less value for money [19]. The reasons for this, as explained by the students, include the advantages of in-person learning such as the ability to interact with lecturers, ability to ask technical questions to both lecturer and peers, and perform group work.

### The unexpected: Substantial proportion of students did not select In-person mode as their 1^st^ choice

Based on one of our objectives, we had hypothesised that a vast majority of students would select In-person mode as their 1^st^ choice. Interestingly, although majority of students did select this learning mode as their 1^st^ choice, this majority was only about 50% of students (Fig 2A). The remaining approximately 50% students selected the other options i.e., Blended (33% students) or fully online modes (14.5% students) as their 1^st^ choices. This 50–50 split contradicts the essence of the students’ petition filed during the pandemic wherein the value of online learning was questioned and put in jeopardy, and it implied that students valued in-person teaching more than online teaching-learning.

There could be several reasons for this deviation from the expected i.e., a large proportion of students not selecting In-person mode as their 1^st^ choice. These could include travel cost incurred when attending in-person sessions, travel distance (time) to university, work commitments etc. Other factors like whether single or multiple sessions are scheduled on the same day can determine whether a student wishes to attend sessions in person or learn online. Also, as deduced from a study during the pandemic, although some students found online learning challenging in the beginning, they quickly adapted to it [24]. Thus, students may have now got accustomed to listening to online lectures in the comforts of their homes, and therefore found blended learning and fully online modes as feasible learning options. Collectively, these aspects fully or partly explain the reason for a substantial proportion of students choosing fully online or blended learning modes as their 1st choices, instead of the expected.

Our data that almost half the student population selected either blended or fully online learning modes as their 1^st^ choice (Fig 2A) should not be surprising. This is because students use technology very widely outside their education in the form of internet and social media. Contextually, how they learn has drastically changed over the past two decades, and this change was accelerated due to the COVID-19 pandemic. An inclination to learn via digital means is natural for today’s generation of students. So, students do expect the use of technology in their learning experience [25]. Moreover, students see benefits in utilizing digital technology and so it has now become an integral part of the university experience [26]. Alongside, it is believed that students do not have patience for lecture-based classes [25]. No wonder, studies have indicated that online education was well-received by the students during the pandemic and a vast majority of them desired online sessions in some form or another [27].

### Concern: Mode of learning (online or in-person) affecting student performance

Despite the apparent benefits of online education, one concern is whether there is or would be a difference in student attainment between students studying online and in-person. In a study conducted during the pandemic, the online cohort of dental students in U.S scored equally well or were more likely to score better than the in-person cohort for the same course in the pre-pandemic quarter [27]. Likewise, the academic performances of undergraduate Psychology students at an Australian university were similar between the two groups of students that took assessments in-person and online [21]. This implies that the mode of teaching and learning, whether online or in-person, may not necessarily determine or influence the academic outcome. As such, online assessments and the format in general have been welcomed by the majority of Bioscience students of a UK university [28]. However, it is pivotal to remember that the influence of learning mode on academic performance can be course dependent. Certain courses and levels of study require the students to gain practical skills, for example, hospital placements in the MBBS programme. In such programmes, if the sessions are not held in-person, the learning of diagnostic and treatment approaches can become limited. As such, the National Health Service at UK has urged the General Practitioners to offer in-person appointments without a prior online or phone triage, as this approach was believed to prevent some patients from accessing the care they need [29].

### The ‘middle ground’: To exercise Blended mode, but with caution

Here, overall, a large proportion of students (43.5%) selected Blended mode as their 2^nd^ choice (Fig 2A). Moreover, students of Language Education, Psychology and Computer Science selected the above mode as their 1^st^ choice (Table 1). Collectively, this indicates that students in general and in certain disciplines do favour blended learning approaches. The reason for this could be that while the students prefer in-person mode, they often have competing interests for their time such as part-time work to support themselves financially or family commitments or social activities. Therefore, they prefer pedagogic approaches that provide flexibility in learning [25]. The concept of blended learning that combine the benefits of both in-person and online teaching had emerged much before the pandemic and it was already being embedded within educational institutions [30]. Some educators even showed that blended learning is tool to enhance student achievement/experience [31–33]. It has been suggested that if blended learning is to be made successful and we want the students to see this approach in a positive light, then it is essential to embed social elements into blended learning to enhance student experience [20].

Despite our overall data showing Blended mode as the next best option to In-person mode (Fig 2A), it is noteworthy that only about a third of males and females, and a third of undergraduates and postgraduates selected the blended mode as their 1^st^ choice (Fig 5B and 5C), implying that the rest of students preferred other modes as their 1^st^ choice. Also, although there were major and significant differences between some degree cohorts in the proportions of students selecting this mode as the 1^st^ choice (Fig 5A), the proportion of students selecting this mode as 1^st^ choice did not differ significantly between males and females and between study levels (Fig 5B and 5C). This implies that the students’ preference for blended learning could be discipline-specific, while biological sex and study level may not have a major influence on the preference for this mode. Therefore, this mode should be deployed with caution because its success and the students’ response to blended learning will depend on the degree cohort, i.e., the subject/content being taught.

As such, significant differences between the mean ranks of the different learning modes (Fig 2B) imply that students’ perceptions towards learning modes differ. This is in line with previous observations where students of Bioscience and Dentistry cohorts showed different perceptions towards using a certain digital technology [2]. Collectively, the data suggests that the same teaching modes cannot be utilised in the same way or same proportion for all programmes. Teaching modes need to be guided by the learning outcomes of the programme and its content for successful outcomes.

### Degree cohort may influence preference for a learning mode

The most frequently selected 1^st^ choice differed between degree cohorts as students (majority) selected either In-person or Blended mode as their 1^st^ choice (Table 1). In Psychology and Computer Science cohorts (In-person mode selected by 42%), the percentage of students selecting Blended and In-person modes was the same or almost the same (Table 1). This suggests that most students of these degree cohorts prefer either of the two learning modes as their 1^st^ choice. The reason for this flexibility of 1^st^ choice between these two learning modes could be related to the nature of the content taught in the lectures of these degree programmes. The content may be such that students may not feel necessary to attend lectures in-person, and instead, may want to revisit those concepts only during in-person tutorials, thereby selecting the Blended mode as their 1^st^ choice.

Regardless, these degree-cohort-based differences in 1^st^ choices together with major differences in the proportions of students selecting Blended mode as the 1^st^ choice as well as last choice across the degree cohorts (Fig 5A) collectively indicate that degree cohort could influence and predict how students wish to learn. For example, here, for In-person mode, Language Education students showed least preference, while Bioscience and Sports & Exercise Science students showed more preference compared to students on other degree cohorts (Fig 3A). For Online-synchronous mode, Sports & Exercise Science students showed more preference compared to students on other degree cohorts (Fig 3B). Also, compared to Language Education students, Sports & Exercise Science students less preferred the Online-mixed-async-sync mode.

In general, there could be several reasons for the discrepancy between cohorts in their preferred mode of learning. One reason could be the content taught on a certain programme. For example, Language Education studies may involve a large amount of text-based learning that could be learnt online and therefore this cohort may prefer in-person mode less (may not need as many in-person sessions) than other cohorts that have some or substantial element of laboratory-based work, hands-on training sessions/workshops, or observational learning. Such sessions usually require in-person presence of students, thereby determining the preferred mode of learning for these students, and a comparatively higher inclination to choose face-to-face learning mode. In other words, a factor that affects students’ choices is whether the content to be learnt and understood is available/accessible off campus. This could partly explain the reason for the blended mode being preferred more by Language Education, Computer Science and Psychology students but preferred less by Sports & Exercise Science and Pharmacy students, compared to other degree cohorts.

### Study level may partly influence preference for a learning mode

Studies have found differences in academic performances of postgraduates and undergraduates [34–36], but how they prefer to learn has not been investigated. Here, we examined the preferences of learning mode in postgraduates and undergraduates. Our data showed that the 1^st^ choice (In-person mode) and last choice (Online-synchronous) of learning modes, as selected by most students were the same across the two study levels. However, between study levels, there were significant differences in the ranking distributions of both In-person and Online-mixed-async-sync modes (Fig 4A and 4B). This indicated differences in students’ preferences towards these learning modes across the two study levels. This is not surprising because students in their 1^st^ year of degree are usually more reliant on guidance given by the academics whereas those in the later years are more independent and therefore may be able to learn well via modes other than in-person.

Collectively, this suggests that study level may at least partly influence and determine students’ preference for certain learning modes i.e., how they wish to learn. For example, here, more undergraduates than postgraduates showed preference for In-person mode (Fig 4A), and less undergraduates than postgraduates showed preference for Online-mixed-async-sync mode (Fig 4B).

### Biological sex is unlikely to determine choice of learning mode

Several studies have investigated on the gender-based differences in academic performance and on online learning outcomes, but have generally shown inconsistent findings [34]. Whether the choices of learning modes differ or not between biological sexes has not been investigated. Here, not only were the 1^st^ and last choices of learning modes (as chosen by the majority) same for both biological sexes (and even in cases when the biological sex was unknown) but the proportions of students (the majority) making these selections were also similar across biological sexes (known or unknown). Moreover, there were no significant differences in the distribution of rankings of the learning modes between biological sexes (known or unknown). This indicates that students’ preferences (based on ranking of the learning modes) are unlikely to be influenced by biological sex.

### Significance of this study and practical implications

To our knowledge, for the first time, we present empirical evidence of the learning mode choices of students for lectures and tutorials while considering whether and how parameters like discipline, study level and biological sex can impact students’ choices of learning modes. Such an investigation is important because the mode of delivering teaching could be amongst the many factors that affect learning and academic performance [37]. Students’ preferred mode of learning can determine their engagement, retention, and attainment in the post-pandemic era.

This study provides an insight into learning mode preferences of university students and thereby adds significantly to the growing body of knowledge in this area. Importantly, our observations step beyond the studies conducted so far and indicate that discipline i.e., subject content and study level could fully or partly determine students’ choice of learning mode. This implies that although a teaching mode can be used to teach on any programme and any level of study, the extent of its success in engaging students and facilitating learning will not be the same. In other words, a teaching mode cannot be utilised in the same way for different programmes and different study levels.

University academics can use this information to design their teaching-learning sessions accordingly. For example, bearing that the students generally prefer face-to-face learning and blended learning is their 2^nd^ choice, academics teaching in disciplines or study levels that require more text-based theoretical understanding/learning can incorporate a larger proportion of online learning and complement this with face-to-face sessions whereas academics that teach in disciplines or study level that encompass more practical and hands-on learning can include smaller proportion of online teaching and a larger proportion of in-person sessions in their programme.

Interestingly, if the same topic/subject is taught to students on the same study level but different programmes, then we need consider the purpose and learning outcomes of the programme before planning to utilise a certain teaching-learning mode to teach that topic. For example, anatomy teaching-learning for a level-4 student studying Biomedical Sciences is usually different to the extent and intensity of anatomy taught to students at the same level on a Medical or Nursing programme. The academics should also think about the duration of the teaching-learning session or day when planning to utilise a certain teaching-learning mode. For example, to aid understanding of threshold concepts, academics can utilise short online sessions, and these could be followed by in-person sessions that promote critical thinking, and collaborative learning through discussions.

Thus, while this data can help the academics in designing teaching sessions for degree programmes, it can also help the university management in formatting strategies, particularly in the climate of paying sky-high energy prices for running university premises when in-person sessions are delivered.

### Limitations of this study, counteracts and considerations for future studies

In this study, data were collected during in-person or online live sessions. Therefore, the data and conclusions are based on the choices of those students who attended these sessions. These conclusions may not fully reflect the choices of students who were absent from these sessions, and therefore, may not completely represent the choices of the entire student population. So, while it important to consider In-person mode as the most preferred, it is equally important to consider the 2^nd^ most preferred mode i.e., Blended mode because it is likely to reflect the choices of students who were absent from the sessions when the data were collected.

Secondly, majority of the undergraduate participants were 1^st^ year university students, which may have influenced the conclusion. As students transition from the 1^st^ year to the 3^rd^ year of an undergraduate degree programme, their preferences of learning modes may alter, likely because of better understanding of academic expectations and gaining more independence in learning. As such, the 1^st^ year undergraduate students often need more help in navigating through the curriculum compared to the more experienced students of later years of a degree programme. To mitigate this, future studies should collect data from students of each year of the undergraduate degree programme and consider both part-time and full-time postgraduate students to obtain a bigger picture of whether and how students’ preferences of learning modes change as they progress from one academic year to another. Sessions could then be designed accordingly.

## Conclusions

Majority of students selected In-person mode as their 1^st^ choice and Online-synchronous as the last choice. For the vast majority, these choices were the same across study levels and biological sexes, but the 1^st^ choice differed between some degree cohorts, that being either In-person mode or Blended mode. The proportion of students preferring In-person mode as their 1^st^ choice was almost equal to those selecting other learning modes combined. For In-person mode, postgraduates and Language Education students showed less preference, while Sports & Exercise Science and Bioscience students showed more preference. For Online-synchronous mode, Sports & Exercise students showed more preference compared to students on other degree cohorts. About 50% of Language Education students selected Blended mode as their 1^st^ choice. Blended mode was preferred less by Sports & Exercise Science and Pharmacy students but more by Language Education, Computer Science and Psychology students. Sports & Exercise Science students showed lesser preference for Online-mixed-asynchronous-synchronous mode compared to language Education students. Undergraduates showed less preference for Online-mixed-asynchronous-synchronous but more preference for In-person mode than postgraduates. We propose that discipline and study level can fully or partly impact students’ preferences of learning mode.

## Supporting information

S1 File Supporting information for all figures and Table 1. (PDF)

## References

[1] Dhawan S. Online Learning: A Panacea in the Time of COVID-19 Crisis. Journal of Educational Technology Systems. 2020;49: 5–22. doi: 10.1177/0047239520934018

[2] Mehta KJ, Miletich I, Detyna M. Content-specific differences in Padlet perception for collaborative learning amongst undergraduate students. Research in Learning Technology. 2021;29. doi: 10.25304/rlt.v29.2551

[3] Petition: Require universities to partially refund tuition fees for 20/21 due to Covid-19. In: Petitions—UK Government and Parliament [Internet]. [cited 15 Feb 2022]. Available: https://petition.parliament.uk/petitions/324762

[4] Class attendance plummets post-Covid. In: Times Higher Education (THE) [Internet]. 9 Jun 2022 [cited 9 Oct 2022]. Available: https://www.timeshighereducation.com/news/class-attendance-plummets-post-covid

[5] reporters THE. Has student attendance recovered post-pandemic? Take our survey. In: Times Higher Education (THE) [Internet]. 26 May 2022 [cited 9 Oct 2022]. Available: https://www.timeshighereducation.com/news/has-student-attendance-recovered-post-pandemic-take-our-survey

[6] Fan S, Trimble A, Kember D, Muir T, Douglas T, Wang Y, et al. Supporting engagement and retention of online and blended-learning students: A qualitative study from an Australian University. Aust Educ Res. 2023; 1–19. doi: 10.1007/s13384-022-00605-5 36684452 PMC9838283

[7] Hollister B, Nair P, Hill-Lindsay S, Chukoskie L. Engagement in Online Learning: Student Attitudes and Behavior During COVID-19. Frontiers in Education. 2022;7. Available: https://www.frontiersin.org/articles/10.3389/feduc.2022.851019

[8] Keis O, Grab C, Schneider A, Öchsner W. Online or face-to-face instruction? A qualitative study on the electrocardiogram course at the University of Ulm to examine why students choose a particular format. BMC Med Educ. 2017;17: 194. doi: 10.1186/s12909-017-1053-6 29121902 PMC5680799

[9] Page J, Meehan-Andrews T, Weerakkody N, Hughes DL, Rathner JA. Student perceptions and learning outcomes of blended learning in a massive first-year core physiology for allied health subjects. Adv Physiol Educ. 2017;41: 44–55. doi: 10.1152/advan.00005.2016 28143822

[10] Asgari S, Trajkovic J, Rahmani M, Zhang W, Lo RC, Sciortino A. An observational study of engineering online education during the COVID-19 pandemic. PLoS One. 2021;16: e0250041. doi: 10.1371/journal.pone.0250041 33857219 PMC8049279

[11] Almossa SY. University students’ perspectives toward learning and assessment during COVID-19. Educ Inf Technol (Dordr). 2021;26: 7163–7181. doi: 10.1007/s10639-021-10554-8 33967588 PMC8090527

[12] Barbagallo MS, Porter JE, Lamunu M. Evaluation of a Blended Online and Digital Learning Mode of Anatomy and Physiology for Undergraduate Nursing Students. Comput Inform Nurs. 2020;38: 633–637. doi: 10.1097/CIN.0000000000000639 32520781

[13] Lischer S, Safi N, Dickson C. Remote learning and students’ mental health during the Covid-19 pandemic: A mixed-method enquiry. Prospects (Paris). 2021; 1–11. doi: 10.1007/s11125-020-09530-w 33424041 PMC7784617

[14] Aucejo EM, French J, Ugalde Araya MP, Zafar B. The impact of COVID-19 on student experiences and expectations: Evidence from a survey. J Public Econ. 2020;191: 104271. doi: 10.1016/j.jpubeco.2020.104271 32873994 PMC7451187

[15] Dinu LM, Dommett EJ, Baykoca A, Mehta KJ, Everett S, Foster JLH, et al. A Case Study Investigating Mental Wellbeing of University Academics during the COVID-19 Pandemic. Education Sciences. 2021;11: 702. doi: 10.3390/educsci11110702

[16] Werner AM, Tibubos AN, Mülder LM, Reichel JL, Schäfer M, Heller S, et al. The impact of lockdown stress and loneliness during the COVID-19 pandemic on mental health among university students in Germany. Sci Rep. 2021;11: 22637. doi: 10.1038/s41598-021-02024-5 34811422 PMC8609027

[17] Kim H-Y. Statistical notes for clinical researchers: Chi-squared test and Fisher’s exact test. Restor Dent Endod. 2017;42: 152–155. doi: 10.5395/rde.2017.42.2.152 28503482 PMC5426219

[18] Mishra P, Pandey CM, Singh U, Keshri A, Sabaretnam M. Selection of Appropriate Statistical Methods for Data Analysis. Ann Card Anaesth. 2019;22: 297–301. doi: 10.4103/aca.ACA_248_18 31274493 PMC6639881

[19] Lomer S, Palmer E. ‘I didn’t know this was actually stuff that could help us, with actually learning’: student perceptions of Active Blended Learning. Teaching in Higher Education. 2021;0: 1–20. doi: 10.1080/13562517.2020.1852202

[20] Mali D, Lim H. How do students perceive face-to-face/blended learning as a result of the Covid-19 pandemic? The International Journal of Management Education. 2021;19: 100552. doi: 10.1016/j.ijme.2021.100552

[21] Kemp N, Grieve R. Face-to-face or face-to-screen? Undergraduates’ opinions and test performance in classroom vs. online learning. Frontiers in Psychology. 2014;5. Available: https://www.frontiersin.org/article/10.3389/fpsyg.2014.01278 25429276 10.3389/fpsyg.2014.01278 PMC4228829

[22] Abdelkader A, Barbagallo MS. The Impact of the COVID-19 Global Pandemic on Undergraduate Nursing Students’ Study of Anatomy and Physiology. Comput Inform Nurs. 2021;40: 278–284. doi: 10.1097/CIN.0000000000000851 34740220 PMC8993758

[23] Selwyn N. Digital downsides: exploring university students’ negative engagements with digital technology. Teaching in Higher Education. 2016;21: 1006–1021. doi: 10.1080/13562517.2016.1213229

[24] Almendingen K, Morseth MS, Gjølstad E, Brevik A, Tørris C. Student’s experiences with online teaching following COVID-19 lockdown: A mixed methods explorative study. PLOS ONE. 2021;16: e0250378. doi: 10.1371/journal.pone.0250378 34464386 PMC8407578

[25] Lashley M, McCleery R. Intensive Laboratory experiences to safely retain experiential learning in the transition to online learning. Ecol Evol. 2020;10: 12613–12619. doi: 10.1002/ece3.6886 33250997 PMC7679533

[26] Henderson M, Selwyn N, Aston R. What works and why? Student perceptions of ‘useful’ digital technology in university teaching and learning. Studies in Higher Education. 2017;42: 1567–1579. doi: 10.1080/03075079.2015.1007946

[27] Zheng M, Bender D, Lyon C. Online learning during COVID-19 produced equivalent or better student course performance as compared with pre-pandemic: empirical evidence from a school-wide comparative study. BMC Med Educ. 2021;21: 495. doi: 10.1186/s12909-021-02909-z 34530828 PMC8443899

[28] Bashir A, Bashir S, Rana K, Lambert P, Vernallis A. Post-COVID-19 Adaptations; the Shifts Towards Online Learning, Hybrid Course Delivery and the Implications for Biosciences Courses in the Higher Education Setting. Frontiers in Education. 2021;6. Available: https://www.frontiersin.org/articles/10.3389/feduc.2021.711619

[29] Iacobucci G. GPs should return to offering face-to-face appointments without prior triage, says NHS. BMJ. 2021;373: n1251. doi: 10.1136/bmj.n1251 33990340

[30] Dziuban C, Graham CR, Moskal PD, Norberg A, Sicilia N. Blended learning: the new normal and emerging technologies. Int J Educ Technol High Educ. 2018;15: 3. doi: 10.1186/s41239-017-0087-5

[31] Bernard RM, Borokhovski E, Schmid RF, Tamim RM, Abrami PC. A meta-analysis of blended learning and technology use in higher education: from the general to the applied. J Comput High Educ. 2014;26: 87–122. doi: 10.1007/s12528-013-9077-3

[32] Liu Q, Peng W, Zhang F, Hu R, Li Y, Yan W. The Effectiveness of Blended Learning in Health Professions: Systematic Review and Meta-Analysis. Journal of Medical Internet Research. 2016;18: e4807. doi: 10.2196/jmir.4807 26729058 PMC4717286

[33] Spanjers IAE, Könings KD, Leppink J, Verstegen DML, de Jong N, Czabanowska K, et al. The promised land of blended learning: Quizzes as a moderator. Educational Research Review. 2015;15: 59–74. doi: 10.1016/j.edurev.2015.05.001

[34] Yu Z. The effects of gender, educational level, and personality on online learning outcomes during the COVID-19 pandemic. International Journal of Educational Technology in Higher Education. 2021;18: 14. doi: 10.1186/s41239-021-00252-3 34778520 PMC8016506

[35] Puddey IB, Mercer A, Carr SE. Relative progress and academic performance of graduate vs undergraduate entrants to an Australian medical school. BMC Med Educ. 2019;19: 159. doi: 10.1186/s12909-019-1584-0 31113431 PMC6530006

[36] Tadese M, Yeshaneh A, Mulu GB. Determinants of good academic performance among university students in Ethiopia: a cross-sectional study. BMC Med Educ. 2022;22: 1–9. doi: 10.1186/s12909-022-03461-0 35606767 PMC9125903

[37] Mehta KJ. Effect of sleep and mood on academic performance—at interface of physiology, psychology, and education. Humanit Soc Sci Commun. 2022;9: 1–13. doi: 10.1057/s41599-021-01031-1

