# Supplementary material for: University students’ preferences of learning modes post COVID-19-associated lockdowns: In-person, online, and blended
Source: PLoS One. 2024 Jul 19;19(7):e0296670. doi: 10.1371/journal.pone.0296670 (PMC11259289; doi:10.1371/journal.pone.0296670)
Supplement: S1 File — (PDF) [file pone.0296670.s001.pdf]

Supporting information

Supporting information for Fig.1

Frequencies

Statistics

Gender_groups

N	Valid	968
	Missing	0

Gender_groups

		Frequency	Percent	Valid Percent	Cumulative Percent
Valid	male	207	21.4	21.4	21.4
	female	497	51.3	51.3	72.7
	unknown biological sex	264	27.3	27.3	100.0
	Total	968	100.0	100.0	

Statistics

Cohort_type

N	Valid	968
	Missing	0

Cohort_type

		Frequency	Percent	Valid Percent	Cumulative Percent
Valid	Bioscience	418	43.2	43.2	43.2
	Psychology	138	14.3	14.3	57.4
	Pharmacy	104	10.7	10.7	68.2
	Computer Sceince	138	14.3	14.3	82.4
	Sports and Exercise Sceince	115	11.9	11.9	94.3
	Language Education	55	5.7	5.7	100.0
	Total	968	100.0	100.0	

Statistics

UG_or_PG

N	Valid	968
	Missing	0

UG_or_PG

		Frequency	Percent	Valid Percent	Cumulative Percent
Valid	Under-graduate level	809	83.6	83.6	83.6
	Post-graduate level	159	16.4	16.4	100.0
	Total	968	100.0	100.0	

Supporting information for Fig.2

Frequencies

Statistics

		Face_to_face	Live_Online	PreRecLectures_LiveTutorials	PreRecLectures_FaceToFaceTutorials
N	Valid	968	968	968	968
	Missing	0	0	0	0

Frequency Table

Face_to_face

		Frequency	Percent	Valid Percent	Cumulative Percent
Valid	1.00	505	52.2	52.2	52.2
	2.00	206	21.3	21.3	73.5
	3.00	131	13.5	13.5	87.0
	4.00	126	13.0	13.0	100.0
	Total	968	100.0	100.0	

Live_Online

		Frequency	Percent	Valid Percent	Cumulative Percent
Valid	1.00	45	4.6	4.6	4.6
	2.00	173	17.9	17.9	22.5
	3.00	252	26.0	26.0	48.6
	4.00	498	51.4	51.4	100.0
	Total	968	100.0	100.0	

PreRecLectures_LiveTutorials

		Frequency	Percent	Valid Percent	Cumulative Percent
Valid	1.00	96	9.9	9.9	9.9
	2.00	165	17.0	17.0	27.0
	3.00	430	44.4	44.4	71.4
	4.00	277	28.6	28.6	100.0
	Total	968	100.0	100.0	

PreRecLectures_FaceToFaceTutorials

		Frequency	Percent	Valid Percent	Cumulative Percent
Valid	1.00	319	33.0	33.0	33.0
	2.00	421	43.5	43.5	76.4
	3.00	160	16.5	16.5	93.0
	4.00	68	7.0	7.0	100.0
	Total	968	100.0	100.0	

Statistics

		Face_to_face	Live_Online	PreRecLectures_LiveTutorials	PreRecLectures_FaceToFaceTutorials
N	Valid	968	968	968	968
	Missing	0	0	0	0
Mean		1.8740	3.2428	2.9174	1.9762
Std. Error of Mean		.03466	.02912	.02959	.02831
Std. Deviation		1.07843	.90611	.92052	.88094

NPar Tests

Descriptive Statistics

	N	Percentiles		
		25th	50th (Median)	75th
FaceToFace	968	1.0000	1.0000	3.0000
LiveOnline	968	3.0000	4.0000	4.0000
PreRecordedLectures_LiveTutorials	968	2.0000	3.0000	4.0000
PreRecordedLectures_FaceToFaceTutorials	968	1.0000	2.0000	2.0000

Friedman Test

Ranks

	Mean Rank
FaceToFace	1.87
LiveOnline	3.24
PreRecordedLectures_LiveTutorials	2.91
PreRecordedLectures_FaceToFaceTutorials	1.97

Test Statistics[a]

N	968
Chi-Square	810.933
df	3
Asymp. Sig.	<.001

a. Friedman Test

Supporting information for Fig.3

Chi-Square Tests

	Value	df	Asymptotic Significance (2-sided)
Pearson Chi-Square	54.186[a]	15	<.001
Likelihood Ratio	52.488	15	<.001
Linear-by-Linear Association	2.777	1	.096
N of Valid Cases	968		

a. 0 cells (.0%) have expected count less than 5. The minimum expected count is 7.16.

Chi-Square Tests

	Value	df	Asymptotic Significance (2-sided)
Pearson Chi-Square	47.558[a]	15	<.001
Likelihood Ratio	45.097	15	<.001
Linear-by-Linear Association	4.776	1	.029
N of Valid Cases	968		

a. 2 cells (8.3%) have expected count less than 5. The minimum expected count is 2.56.

Supporting information for Fig.4

Chi-Square Tests

	Value	df	Asymptotic Significance (2-sided)
Pearson Chi-Square	12.972[a]	3	.005
Likelihood Ratio	11.776	3	.008
Linear-by-Linear Association	5.457	1	.019
N of Valid Cases	968		

a. 0 cells (.0%) have expected count less than 5. The minimum expected count is 20.70.

Chi-Square Tests

	Value	df	Asymptotic Significance (2-sided)
Pearson Chi-Square	11.231[a]	3	.011
Likelihood Ratio	11.354	3	.010
Linear-by-Linear Association	10.792	1	.001
N of Valid Cases	968		

a. 0 cells (.0%) have expected count less than 5. The minimum expected count is 15.77.

Supporting information for Fig.5

Frequencies

Bioscience only.sav

Statistics

PreRecLectures_FaceToFaceTutorials

N	Valid	418
	Missing	0

PreRecLectures_FaceToFaceTutorials

		Frequency	Percent	Valid Percent	Cumulative Percent
Valid	1.00	131	31.3	31.3	31.3
	2.00	192	45.9	45.9	77.3
	3.00	74	17.7	17.7	95.0
	4.00	21	5.0	5.0	100.0
	Total	418	100.0	100.0	

Frequencies

computer science only.sav

Statistics

PreRecLectures_FaceToFaceTutorials

N	Valid	138
	Missing	0

PreRecLectures_FaceToFaceTutorials

		Frequency	Percent	Valid Percent	Cumulative Percent
Valid	1.00	59	42.8	42.8	42.8
	2.00	54	39.1	39.1	81.9
	3.00	15	10.9	10.9	92.8
	4.00	10	7.2	7.2	100.0
	Total	138	100.0	100.0	

Frequencies

Psychology only.sav

Statistics

PreRecLectures_FaceToFaceTutorials

N	Valid	138
	Missing	0

PreRecLectures_FaceToFaceTutorials

		Frequency	Percent	Valid Percent	Cumulative Percent
Valid	1.00	58	42.0	42.0	42.0
	2.00	53	38.4	38.4	80.4
	3.00	16	11.6	11.6	92.0
	4.00	11	8.0	8.0	100.0
	Total	138	100.0	100.0	

Frequencies

sports only.sav

Statistics

PreRecLectures_FaceToFaceTutorials

N	Valid	115
	Missing	0

PreRecLectures_FaceToFaceTutorials

		Frequency	Percent	Valid Percent	Cumulative Percent
Valid	1.00	14	12.2	12.2	12.2
	2.00	52	45.2	45.2	57.4
	3.00	37	32.2	32.2	89.6
	4.00	12	10.4	10.4	100.0
	Total	115	100.0	100.0	

Frequencies

Language only.sav

Statistics

PreRecLectures_FaceToFaceTutorials

N	Valid	55
	Missing	0

PreRecLectures_FaceToFaceTutorials

		Frequency	Percent	Valid Percent	Cumulative Percent
Valid	1.00	27	49.1	49.1	49.1
	2.00	20	36.4	36.4	85.5
	3.00	7	12.7	12.7	98.2
	4.00	1	1.8	1.8	100.0
	Total	55	100.0	100.0	

Frequencies

Pharmacy only.sav

Statistics

PreRecLectures_FaceToFaceTutorials

N	Valid	104
	Missing	0

PreRecLectures_FaceToFaceTutorials

		Frequency	Percent	Valid Percent	Cumulative Percent
Valid	1.00	30	28.8	28.8	28.8
	2.00	50	48.1	48.1	76.9
	3.00	11	10.6	10.6	87.5
	4.00	13	12.5	12.5	100.0
	Total	104	100.0	100.0	

Frequencies

females only.sav

Statistics

PreRecLectures_FaceToFaceTutorials

N	Valid	497
	Missing	0

PreRecLectures_FaceToFaceTutorials

		Frequency	Percent	Valid Percent	Cumulative Percent
Valid	1.00	181	36.4	36.4	36.4
	2.00	194	39.0	39.0	75.5
	3.00	89	17.9	17.9	93.4
	4.00	33	6.6	6.6	100.0
	Total	497	100.0	100.0	

Frequencies

Males only.sav

Statistics

PreRecLectures_FaceToFaceTutorials

N	Valid	207
	Missing	0

PreRecLectures_FaceToFaceTutorials

		Frequency	Percent	Valid Percent	Cumulative Percent
Valid	1.00	64	30.9	30.9	30.9
	2.00	97	46.9	46.9	77.8
	3.00	29	14.0	14.0	91.8
	4.00	17	8.2	8.2	100.0
	Total	207	100.0	100.0	

Frequencies

Unknown sex only.sav

Statistics

PreRecLectures_FaceToFaceTutorials

N	Valid	264
	Missing	0

PreRecLectures_FaceToFaceTutorials

		Frequency	Percent	Valid Percent	Cumulative Percent
Valid	1.00	74	28.0	28.0	28.0
	2.00	130	49.2	49.2	77.3
	3.00	42	15.9	15.9	93.2
	4.00	18	6.8	6.8	100.0
	Total	264	100.0	100.0	

Frequencies UG only.sav

Statistics

PreRecLectures_FaceToFaceTutorials

N	Valid	809
	Missing	0

PreRecLectures_FaceToFaceTutorials

		Frequency	Percent	Valid Percent	Cumulative Percent
Valid	1.00	262	32.4	32.4	32.4
	2.00	351	43.4	43.4	75.8
	3.00	142	17.6	17.6	93.3
	4.00	54	6.7	6.7	100.0
	Total	809	100.0	100.0	

Frequencies
PG.sav

Statistics

PreRecLectures_FaceToFaceTutorials

N	Valid	159
	Missing	0

PreRecLectures_FaceToFaceTutorials

		Frequency	Percent	Valid Percent	Cumulative Percent
Valid	1.00	57	35.8	35.8	35.8
	2.00	70	44.0	44.0	79.9
	3.00	18	11.3	11.3	91.2
	4.00	14	8.8	8.8	100.0
	Total	159	100.0	100.0	

Supporting information for Table 1

Frequencies

Bioscience only.sav

Statistics

		Face_to_face	Live_Online	PreRecLectures_LiveTutorials	PreRecLectures_FaceToFaceTutorials
N	Valid	418	418	418	418
	Missing	0	0	0	0

Frequency Table

Face_to_face

		Frequency	Percent	Valid Percent	Cumulative Percent
Valid	1.00	239	57.2	57.2	57.2
	2.00	80	19.1	19.1	76.3
	3.00	52	12.4	12.4	88.8
	4.00	47	11.2	11.2	100.0
	Total	418	100.0	100.0	

Live_Online

		Frequency	Percent	Valid Percent	Cumulative Percent
Valid	1.00	11	2.6	2.6	2.6
	2.00	79	18.9	18.9	21.5
	3.00	120	28.7	28.7	50.2
	4.00	208	49.8	49.8	100.0
	Total	418	100.0	100.0	

PreRecLectures_LiveTutorials

		Frequency	Percent	Valid Percent	Cumulative Percent
Valid	1.00	34	8.1	8.1	8.1
	2.00	65	15.6	15.6	23.7
	3.00	176	42.1	42.1	65.8
	4.00	143	34.2	34.2	100.0
	Total	418	100.0	100.0	

PreRecLectures_FaceToFaceTutorials

		Frequency	Percent	Valid Percent	Cumulative Percent
Valid	1.00	131	31.3	31.3	31.3
	2.00	192	45.9	45.9	77.3
	3.00	74	17.7	17.7	95.0
	4.00	21	5.0	5.0	100.0
	Total	418	100.0	100.0	

Frequencies

Psychology only.sav

Statistics

		Face_to_face	Live_Online	PreRecLectures_LiveTutorials	PreRecLectures_FaceToFaceTutorials
N	Valid	138	138	138	138
	Missing	0	0	0	0

Frequency Table

Face_to_face

		Frequency	Percent	Valid Percent	Cumulative Percent
Valid	1.00	58	42.0	42.0	42.0
	2.00	46	33.3	33.3	75.4
	3.00	16	11.6	11.6	87.0
	4.00	18	13.0	13.0	100.0
	Total	138	100.0	100.0	

Live_Online

		Frequency	Percent	Valid Percent	Cumulative Percent
Valid	1.00	8	5.8	5.8	5.8
	2.00	16	11.6	11.6	17.4
	3.00	31	22.5	22.5	39.9
	4.00	83	60.1	60.1	100.0
	Total	138	100.0	100.0	

PreRecLectures_LiveTutorials

		Frequency	Percent	Valid Percent	Cumulative Percent
Valid	1.00	14	10.1	10.1	10.1
	2.00	23	16.7	16.7	26.8
	3.00	75	54.3	54.3	81.2
	4.00	26	18.8	18.8	100.0
	Total	138	100.0	100.0	

PreRecLectures_FaceToFaceTutorials

		Frequency	Percent	Valid Percent	Cumulative Percent
Valid	1.00	58	42.0	42.0	42.0
	2.00	53	38.4	38.4	80.4
	3.00	16	11.6	11.6	92.0
	4.00	11	8.0	8.0	100.0
	Total	138	100.0	100.0	

Frequencies

Pharmacy only.sav

Statistics

		Face_to_face	Live_Online	PreRecLectures_LiveTutorials	PreRecLectures_FaceToFaceTutorials
N	Valid	104	104	104	104
	Missing	0	0	0	0

Frequency Table

Face_to_face

		Frequency	Percent	Valid Percent	Cumulative Percent
Valid	1.00	51	49.0	49.0	49.0
	2.00	15	14.4	14.4	63.5
	3.00	26	25.0	25.0	88.5
	4.00	12	11.5	11.5	100.0
	Total	104	100.0	100.0	

Live_Online

		Frequency	Percent	Valid Percent	Cumulative Percent
Valid	1.00	5	4.8	4.8	4.8
	2.00	18	17.3	17.3	22.1
	3.00	19	18.3	18.3	40.4
	4.00	62	59.6	59.6	100.0
	Total	104	100.0	100.0	

PreRecLectures_LiveTutorials

		Frequency	Percent	Valid Percent	Cumulative Percent
Valid	1.00	18	17.3	17.3	17.3
	2.00	21	20.2	20.2	37.5
	3.00	48	46.2	46.2	83.7
	4.00	17	16.3	16.3	100.0
	Total	104	100.0	100.0	

PreRecLectures_FaceToFaceTutorials

		Frequency	Percent	Valid Percent	Cumulative Percent
Valid	1.00	30	28.8	28.8	28.8
	2.00	50	48.1	48.1	76.9
	3.00	11	10.6	10.6	87.5
	4.00	13	12.5	12.5	100.0
	Total	104	100.0	100.0	

Frequencies

computer science only.sav

Statistics

		Face_to_face	Live_Online	PreRecLectures_LiveTutorials	PreRecLectures_FaceToFaceTutorials
N	Valid	138	138	138	138
	Missing	0	0	0	0

Frequency Table

Face_to_face

		Frequency	Percent	Valid Percent	Cumulative Percent
Valid	1.00	58	42.0	42.0	42.0
	2.00	36	26.1	26.1	68.1
	3.00	21	15.2	15.2	83.3
	4.00	23	16.7	16.7	100.0
	Total	138	100.0	100.0	

Live_Online

		Frequency	Percent	Valid Percent	Cumulative Percent
Valid	1.00	5	3.6	3.6	3.6
	2.00	19	13.8	13.8	17.4
	3.00	37	26.8	26.8	44.2
	4.00	77	55.8	55.8	100.0
	Total	138	100.0	100.0	

PreRecLectures_LiveTutorials

		Frequency	Percent	Valid Percent	Cumulative Percent
Valid	1.00	16	11.6	11.6	11.6
	2.00	29	21.0	21.0	32.6
	3.00	65	47.1	47.1	79.7
	4.00	28	20.3	20.3	100.0
	Total	138	100.0	100.0	

PreRecLectures_FaceToFaceTutorials

		Frequency	Percent	Valid Percent	Cumulative Percent
Valid	1.00	59	42.8	42.8	42.8
	2.00	54	39.1	39.1	81.9
	3.00	15	10.9	10.9	92.8
	4.00	10	7.2	7.2	100.0
	Total	138	100.0	100.0	

Frequencies

sports only.sav

Statistics

		Face_to_face	Live_Online	PreRecLectures_LiveTutorials	PreRecLectures_FaceToFaceTutorials
N	Valid	115	115	115	115
	Missing	0	0	0	0

Frequency Table

Face_to_face

		Frequency	Percent	Valid Percent	Cumulative Percent
Valid	1.00	78	67.8	67.8	67.8
	2.00	15	13.0	13.0	80.9
	3.00	7	6.1	6.1	87.0
	4.00	15	13.0	13.0	100.0
	Total	115	100.0	100.0	

Live_Online

		Frequency	Percent	Valid Percent	Cumulative Percent
Valid	1.00	13	11.3	11.3	11.3
	2.00	35	30.4	30.4	41.7
	3.00	28	24.3	24.3	66.1
	4.00	39	33.9	33.9	100.0
	Total	115	100.0	100.0	

PreRecLectures_LiveTutorials

		Frequency	Percent	Valid Percent	Cumulative Percent
Valid	1.00	10	8.7	8.7	8.7
	2.00	13	11.3	11.3	20.0
	3.00	43	37.4	37.4	57.4
	4.00	49	42.6	42.6	100.0
	Total	115	100.0	100.0	

PreRecLectures_FaceToFaceTutorials

		Frequency	Percent	Valid Percent	Cumulative Percent
Valid	1.00	14	12.2	12.2	12.2
	2.00	52	45.2	45.2	57.4
	3.00	37	32.2	32.2	89.6
	4.00	12	10.4	10.4	100.0
	Total	115	100.0	100.0	

Frequencies

Language only.sav

Statistics

		Face_to_face	Live_Online	PreRecLectures_LiveTutorials	PreRecLectures_FaceToFaceTutorials
N	Valid	55	55	55	55
	Missing	0	0	0	0

Frequency Table

Face_to_face

		Frequency	Percent	Valid Percent	Cumulative Percent
Valid	1.00	21	38.2	38.2	38.2
	2.00	14	25.5	25.5	63.6
	3.00	9	16.4	16.4	80.0
	4.00	11	20.0	20.0	100.0
	Total	55	100.0	100.0	

Live_Online

		Frequency	Percent	Valid Percent	Cumulative Percent
Valid	1.00	3	5.5	5.5	5.5
	2.00	6	10.9	10.9	16.4
	3.00	17	30.9	30.9	47.3
	4.00	29	52.7	52.7	100.0
	Total	55	100.0	100.0	

PreRecLectures_LiveTutorials

		Frequency	Percent	Valid Percent	Cumulative Percent
Valid	1.00	4	7.3	7.3	7.3
	2.00	14	25.5	25.5	32.7
	3.00	23	41.8	41.8	74.5
	4.00	14	25.5	25.5	100.0
	Total	55	100.0	100.0	

PreRecLectures_FaceToFaceTutorials

		Frequency	Percent	Valid Percent	Cumulative Percent
Valid	1.00	27	49.1	49.1	49.1
	2.00	20	36.4	36.4	85.5
	3.00	7	12.7	12.7	98.2
	4.00	1	1.8	1.8	100.0
	Total	55	100.0	100.0	

Data for ordinal regression considering degree cohorts.

PLUM - Ordinal Regression

Case Processing Summary

		N	Marginal Percentage
F2F_Lec_Tuto	1.00	505	52.2%
	2.00	206	21.3%
	3.00	131	13.5%
	4.00	126	13.0%
Cohort_type	Bioscience	418	43.2%
	Psychology	138	14.3%
	Pharmacy	104	10.7%
	Computer Sceince	138	14.3%
	Sports and Exercise Sceince	115	11.9%
	Language Education	55	5.7%
Valid		968	100.0%
Missing		169	
Total		1137	

Model Fitting Information

Model	-2 Log Likelihood	Chi-Square	df	Sig.
Intercept Only	135.885			
Final	109.962	25.923	5	<.001

Link function: Logit.

Goodness-of-Fit

	Chi-Square	df	Sig.
Pearson	28.387	10	.002
Deviance	26.564	10	.003

Link function: Logit.

Pseudo R-Square

Cox and Snell	.026
Nagelkerke	.029
McFadden	.011

Link function: Logit.

Cell Information

Frequency

Cohort_type		F2F_Lec_Tuto 1.00	2.00	3.00	4.00
Bioscience	Observed	239	80	52	47
	Expected	236.835	85.983	50.069	45.114
	Pearson Residual	.214	-.724	.291	.297
Psychology	Observed	58	46	16	18
	Expected	64.109	31.441	21.172	21.278
	Pearson Residual	-1.043	2.955	-1.222	-.773
Pharmacy	Observed	51	15	26	12
	Expected	49.013	23.591	15.724	15.673
	Pearson Residual	.390	-2.012	2.813	-1.007
Computer Sceince	Observed	58	36	21	23
	Expected	59.727	31.959	22.621	23.693
	Pearson Residual	-.297	.815	-.373	-.156
Sports and Exercise Sceince	Observed	78	15	7	15
	Expected	75.821	20.078	10.412	8.688
	Pearson Residual	.429	-1.247	-1.109	2.227
Language Education	Observed	21	14	9	11
	Expected	21.425	12.863	9.787	10.925
	Pearson Residual	-.117	.362	-.278	.026

Link function: Logit.

Test of Parallel Lines[a]

Model	-2 Log Likelihood	Chi-Square	df	Sig.
Null Hypothesis	109.962			
General	83.397	26.564	10	.003

The null hypothesis states that the location parameters (slope coefficients) are the same across response categories.

a. Link function: Logit.

PLUM - Ordinal Regression

Case Processing Summary

		N	Marginal Percentage
PreRecordedLecturesF2Ftutorials	1.00	319	33.0%
	2.00	421	43.5%
	3.00	160	16.5%
	4.00	68	7.0%
Cohort_type	Bioscience	418	43.2%
	Psychology	138	14.3%
	Pharmacy	104	10.7%
	Computer Sceince	138	14.3%
	Sports and Exercise Sceince	115	11.9%
	Language Education	55	5.7%
Valid		968	100.0%
Missing		169	
Total		1137	

Model Fitting Information

Model	-2 Log Likelihood	Chi-Square	df	Sig.
Intercept Only	148.941			
Final	101.105	47.835	5	<.001

Link function: Logit.

Goodness-of-Fit

	Chi-Square	df	Sig.
Pearson	20.504	10	.025
Deviance	20.011	10	.029

Link function: Logit.

Pseudo R-Square

Cox and Snell	.048
Nagelkerke	.053
McFadden	.020

Link function: Logit.

Cell Information

Frequency

Cohort_type		PreRecordedLecturesF2Ftutorials 1.00	2.00	3.00	4.00
Bioscience	Observed	131	192	74	21
	Expected	133.622	188.549	68.237	27.592
	Pearson Residual	-.275	.339	.763	-1.299
Psychology	Observed	58	53	16	11
	Expected	55.059	58.939	17.427	6.575
	Pearson Residual	.511	-1.022	-.366	1.769
Pharmacy	Observed	30	50	11	13
	Expected	30.273	47.317	18.629	7.782
	Pearson Residual	-.059	.528	-1.951	1.945
Computer Sceince	Observed	59	54	15	10
	Expected	56.900	58.176	16.687	6.237
	Pearson Residual	.363	-.720	-.441	1.542
Sports and Exercise Sceince	Observed	14	52	37	12
	Expected	18.142	47.716	31.824	17.319
	Pearson Residual	-1.060	.811	1.079	-1.387
Language Education	Observed	27	20	7	1
	Expected	26.645	21.234	5.244	1.877
	Pearson Residual	.096	-.342	.806	-.652

Link function: Logit.

Test of Parallel Lines[a]

Model	-2 Log Likelihood	Chi-Square	df	Sig.
Null Hypothesis	101.105			
General	81.094	20.011	10	.029

The null hypothesis states that the location parameters (slope coefficients) are the same across response categories.

a. Link function: Logit.

PLUM - Ordinal Regression

Case Processing Summary

		N	Marginal Percentage
Liveonline	1.00	45	4.6%
	2.00	173	17.9%
	3.00	252	26.0%
	4.00	498	51.4%
Cohort_type	Bioscience	418	43.2%
	Psychology	138	14.3%
	Pharmacy	104	10.7%
	Computer Sceince	138	14.3%
	Sports and Exercise Sceince	115	11.9%
	Language Education	55	5.7%
Valid		968	100.0%
Missing		169	
Total		1137	

Model Fitting Information

Model	-2 Log Likelihood	Chi-Square	df	Sig.
Intercept Only	125.054			
Final	94.178	30.876	5	<.001

Link function: Logit.

Goodness-of-Fit

	Chi-Square	df	Sig.
Pearson	14.070	10	.170
Deviance	14.220	10	.163

Link function: Logit.

Pseudo R-Square

Cox and Snell	.031
Nagelkerke	.035
McFadden	.014

Link function: Logit.

Cell Information

Frequency

Cohort_type		Liveonline 1.00	2.00	3.00	4.00
Bioscience	Observed	11	79	120	208
	Expected	18.717	74.947	111.995	212.341
	Pearson Residual	-1.825	.517	.884	-.425
Psychology	Observed	8	16	31	83
	Expected	4.418	18.940	32.661	81.981
	Pearson Residual	1.732	-.727	-.333	.177
Pharmacy	Observed	5	18	19	62
	Expected	3.605	15.235	25.457	59.704
	Pearson Residual	.748	.767	-1.473	.455
Computer Sceince	Observed	5	19	37	77
	Expected	4.969	20.848	34.297	77.886
	Pearson Residual	.014	-.439	.532	-.152
Sports and Exercise Sceince	Observed	13	35	28	39
	Expected	11.175	34.676	33.477	35.672
	Pearson Residual	.575	.066	-1.124	.671
Language Education	Observed	3	6	17	29
	Expected	2.154	8.886	14.105	29.855
	Pearson Residual	.588	-1.057	.894	-.231

Link function: Logit.

Test of Parallel Lines[a]

Model	-2 Log Likelihood	Chi-Square	df	Sig.
Null Hypothesis	94.178			
General	79.958	14.220	10	.163

The null hypothesis states that the location parameters (slope coefficients) are the same across response categories.

a. Link function: Logit.

PLUM - Ordinal Regression

Case Processing Summary

		N	Marginal Percentage
PreRecordedLecturesLivetutorials	1.00	96	9.9%
	2.00	165	17.0%
	3.00	430	44.4%
	4.00	277	28.6%
Cohort_type	Bioscience	418	43.2%
	Psychology	138	14.3%
	Pharmacy	104	10.7%
	Computer Sceince	138	14.3%
	Sports and Exercise Sceince	115	11.9%
	Language Education	55	5.7%
Valid		968	100.0%
Missing		169	
Total		1137	

Model Fitting Information

Model	-2 Log Likelihood	Chi-Square	df	Sig.
Intercept Only	129.587			
Final	95.875	33.712	5	<.001

Link function: Logit.

Goodness-of-Fit

	Chi-Square	df	Sig.
Pearson	12.306	10	.265
Deviance	12.096	10	.279

Link function: Logit.

Pseudo R-Square

Cox and Snell	.034
Nagelkerke	.037
McFadden	.014

Link function: Logit.

Cell Information

Frequency

Cohort_type		PreRecordedLecturesLivetutorials 1.00	2.00	3.00	4.00
Bioscience	Observed	34	65	176	143
	Expected	32.305	60.454	187.482	137.759
	Pearson Residual	.311	.632	-1.129	.545
Psychology	Observed	14	23	75	26
	Expected	16.194	26.811	62.364	32.631
	Pearson Residual	-.580	-.820	2.161	-1.328
Pharmacy	Observed	18	21	48	17
	Expected	16.715	24.334	44.548	18.404
	Pearson Residual	.343	-.772	.684	-.361
Computer Sceince	Observed	16	29	65	28
	Expected	17.769	28.430	61.733	30.068
	Pearson Residual	-.450	.120	.559	-.426
Sports and Exercise Sceince	Observed	10	13	43	49
	Expected	6.636	13.206	48.923	46.236
	Pearson Residual	1.345	-.060	-1.117	.526
Language Education	Observed	4	14	23	14
	Expected	6.151	10.354	24.942	13.552
	Pearson Residual	-.920	1.257	-.526	.140

Link function: Logit.

Test of Parallel Lines[a]

Model	-2 Log Likelihood	Chi-Square	df	Sig.
Null Hypothesis	95.875			
General	83.779	12.096	10	.279

The null hypothesis states that the location parameters (slope coefficients) are the same across response categories.

a. Link function: Logit.

Generalized Linear Models

Model Information

Dependent Variable	Liveonline[a]
Probability Distribution	Multinomial
Link Function	Cumulative logit

a. The procedure applies the cumulative link function to the dependent variable values in ascending order.

Case Processing Summary

	N	Percent
Included	968	85.1%
Excluded	169	14.9%
Total	1137	100.0%

Categorical Variable Information

			N	Percent
Dependent Variable	Liveonline	1.00	45	4.6%
		2.00	173	17.9%
		3.00	252	26.0%
		4.00	498	51.4%
		Total	968	100.0%
Factor	Cohort_type	Bioscience	418	43.2%
		Psychology	138	14.3%
		Pharmacy	104	10.7%
		Computer Sceince	138	14.3%
		Sports and Exercise Sceince	115	11.9%
		Language Education	55	5.7%
		Total	968	100.0%

Goodness of Fit[a]

	Value	df	Value/df
Deviance	14.220	10	1.422
Scaled Deviance	14.220	10	
Pearson Chi-Square	14.070	10	1.407
Scaled Pearson Chi-Square	14.070	10	
Log Likelihood[b]	-47.089		
Akaike's Information Criterion (AIC)	110.178		
Finite Sample Corrected AIC (AICC)	110.328		

Bayesian Information Criterion (BIC)	149.180		
Consistent AIC (CAIC)	157.180		

Dependent Variable: Liveonline

Model: (Threshold), Cohort_type[a]

a. Information criteria are in smaller-is-better form.

b. The full log likelihood function is displayed and used in computing information criteria.

Omnibus Test[a]

Likelihood Ratio Chi-Square	df	Sig.
30.876	5	<.001

Dependent Variable: Liveonline

Model: (Threshold), Cohort_type[a]

a. Compares the fitted model against the thresholds-only model.

Tests of Model Effects

	Type III		
Source	Likelihood Ratio Chi-Square	df	Sig.
Cohort_type	30.876[a]	5	<.001

Dependent Variable: Liveonline

Model: (Threshold), Cohort_type

a. The validity of the likelihood ratio chi-square is uncertain because log-likelihood convergence was not achieved for the constrained model. Results shown are based on the last iteration.

Parameter Estimates

Parameter		B	Std. Error	95% Wald Confidence Interval		Hypothesis Test			Exp(B)	95% Wald Confidence Interval for Exp(B)	
				Lower	Upper	Wald Chi-Square	df	Sig.		Lower	Upper
Threshold	[Liveonline=1.00]	-3.200	.2927	-3.774	-2.626	119.510	1	.000	.041	.023	.072
	[Liveonline=2.00]	-1.382	.2597	-1.891	-.873	28.318	1	<.001	.251	.151	.418
	[Liveonline=3.00]	-.172	.2551	-.672	.328	.453	1	.501	.842	.511	1.389
[Cohort_type=1.00]		-.140	.2703	-.669	.390	.267	1	.605	.870	.512	1.477
[Cohort_type=2.00]		.209	.3057	-.390	.808	.468	1	.494	1.233	.677	2.244
[Cohort_type=3.00]		.127	.3211	-.503	.756	.156	1	.693	1.135	.605	2.130
[Cohort_type=4.00]		.087	.3025	-.506	.680	.083	1	.773	1.091	.603	1.974
[Cohort_type=5.00]		-.971	.3084	-1.575	-.366	9.911	1	.002	.379	.207	.693
[Cohort_type=6.00]		0[a]	.	.	.	.	.	.	1	.	.
(Scale)		1[b]									

Dependent Variable: Liveonline

Model: (Threshold), Cohort_type

a. Set to zero because this parameter is redundant.

b. Fixed at the displayed value.

Generalized Linear Models

Model Information

Dependent Variable	PreRecordedLecturesLivetutorials[a]
Probability Distribution	Multinomial
Link Function	Cumulative logit

a. The procedure applies the cumulative link function to the dependent variable values in ascending order.

Case Processing Summary

	N	Percent
Included	968	85.1%
Excluded	169	14.9%
Total	1137	100.0%

Categorical Variable Information

			N	Percent
Dependent Variable	PreRecordedLecturesLivetutorials	1.00	96	9.9%
		2.00	165	17.0%
		3.00	430	44.4%
		4.00	277	28.6%
		Total	968	100.0%
Factor	Cohort_type	Bioscience	418	43.2%
		Psychology	138	14.3%
		Pharmacy	104	10.7%
		Computer Sceince	138	14.3%
		Sports and Exercise Sceince	115	11.9%
		Language Education	55	5.7%
		Total	968	100.0%

Goodness of Fit[a]

	Value	df	Value/df
Deviance	12.096	10	1.210
Scaled Deviance	12.096	10	
Pearson Chi-Square	12.306	10	1.231
Scaled Pearson Chi-Square	12.306	10	
Log Likelihood[b]	-47.937		

Akaike's Information Criterion (AIC)	111.875		
Finite Sample Corrected AIC (AICC)	112.025		
Bayesian Information Criterion (BIC)	150.877		
Consistent AIC (CAIC)	158.877		

Dependent Variable: PreRecordedLecturesLivetutorials

Model: (Threshold), Cohort_type[a]

a. Information criteria are in smaller-is-better form.

b. The full log likelihood function is displayed and used in computing information criteria.

Omnibus Test[a]

Likelihood Ratio Chi-Square	df	Sig.
33.712	5	<.001

Dependent Variable: PreRecordedLecturesLivetutorials

Model: (Threshold), Cohort_type[a]

a. Compares the fitted model against the thresholds-only model.

Tests of Model Effects

	Type III		
Source	Likelihood Ratio Chi-Square	df	Sig.
Cohort_type	33.712[a]	5	<.001

Dependent Variable: PreRecordedLecturesLivetutorials

Model: (Threshold), Cohort_type

a. The validity of the likelihood ratio chi-square is uncertain because log-likelihood convergence was not achieved for the constrained model. Results shown are based on the last iteration.

Parameter Estimates

				95% Wald Confidence Interval		Hypothesis Test				95% Wald Confidence Interval for Exp(B)	
Parameter		B	Std. Error	Lower	Upper	Wald Chi-Square	df	Sig.	Exp(B)	Lower	Upper
Threshold	[PreRecordedLecturesLivetutorials=1.00]	-2.072	.2614	-2.584	-1.560	62.849	1	<.001	.126	.075	.210
	[PreRecordedLecturesLivetutorials=2.00]	-.847	.2494	-1.336	-.358	11.526	1	<.001	.429	.263	.699
	[PreRecordedLecturesLivetutorials=3.00]	1.118	.2514	.625	1.611	19.776	1	<.001	3.058	1.869	5.006
[Cohort_type=1.00]		.408	.2636	-.109	.924	2.393	1	.122	1.503	.897	2.520
[Cohort_type=2.00]		-.054	.2900	-.623	.514	.035	1	.851	.947	.537	1.672
[Cohort_type=3.00]		-.419	.3050	-1.017	.179	1.889	1	.169	.658	.362	1.195
[Cohort_type=4.00]		-.160	.2912	-.731	.411	.303	1	.582	.852	.482	1.508
[Cohort_type=5.00]		.721	.3055	.122	1.320	5.570	1	.018	2.056	1.130	3.742
[Cohort_type=6.00]		0[a]	.	.	.	.	.	.	1	.	.
(Scale)		1[b]									

Dependent Variable: PreRecordedLecturesLivetutorials
Model: (Threshold), Cohort_type

a. Set to zero because this parameter is redundant.

b. Fixed at the displayed value.

Data for ordinal regression considering study levels.

PLUM - Ordinal Regression

Case Processing Summary

		N	Marginal Percentage
F2F_Lec_Tuto	1.00	505	52.2%
	2.00	206	21.3%
	3.00	131	13.5%
	4.00	126	13.0%
UG_or_PG	Under-graduate level	809	83.6%
	Post-graduate level	159	16.4%
Valid		968	100.0%
Missing		169	
Total		1137	

Model Fitting Information

Model	-2 Log Likelihood	Chi-Square	df	Sig.
Intercept Only	45.843			
Final	40.534	5.310	1	.021

Link function: Logit.

Goodness-of-Fit

	Chi-Square	df	Sig.
Pearson	6.678	2	.035
Deviance	6.467	2	.039

Link function: Logit.

Pseudo R-Square

Cox and Snell	.005
Nagelkerke	.006
McFadden	.002

Link function: Logit.

Parameter Estimates

		Estimate	Std. Error	Wald	df	Sig.	95% Confidence Interval Lower Bound	95% Confidence Interval Upper Bound
Threshold	[F2F_Lec_Tuto = 1.00]	-.225	.148	2.312	1	.128	-.516	.065
	[F2F_Lec_Tuto = 2.00]	.711	.150	22.527	1	<.001	.418	1.005
	[F2F_Lec_Tuto = 3.00]	1.598	.161	98.689	1	<.001	1.282	1.913
Location	[UG_or_PG=1.00]	-.372	.160	5.398	1	.020	-.686	-.058
	[UG_or_PG=2.00]	0[a]	.	.	0	.	.	.

Link function: Logit.

a. This parameter is set to zero because it is redundant.

Cell Information

Frequency

UG_or_PG		F2F_Lec_Tuto 1.00	2.00	3.00	4.00
Under-graduate level	Observed	433	177	96	103
	Expected	434.179	170.292	105.500	99.029
	Pearson Residual	-.083	.579	-.992	.426
Post-graduate level	Observed	72	29	35	23
	Expected	70.585	36.058	25.594	26.763
	Pearson Residual	.226	-1.337	2.030	-.798

Link function: Logit.

Test of Parallel Lines[a]

Model	-2 Log Likelihood	Chi-Square	df	Sig.
Null Hypothesis	40.534			
General	34.067	6.467	2	.039

The null hypothesis states that the location parameters (slope coefficients) are the same across response categories.

a. Link function: Logit.

PLUM - Ordinal Regression

Case Processing Summary

		N	Marginal Percentage
Liveonline	1.00	45	4.6%
	2.00	173	17.9%
	3.00	252	26.0%
	4.00	498	51.4%
UG_or_PG	Under-graduate level	809	83.6%
	Post-graduate level	159	16.4%
Valid		968	100.0%
Missing		169	
Total		1137	

Model Fitting Information

Model	-2 Log Likelihood	Chi-Square	df	Sig.
Intercept Only	35.645			
Final	33.571	2.075	1	.150

Link function: Logit.

Goodness-of-Fit

	Chi-Square	df	Sig.
Pearson	.968	2	.616
Deviance	.918	2	.632

Link function: Logit.

Pseudo R-Square

Cox and Snell	.002
Nagelkerke	.002
McFadden	.001

Link function: Logit.

Parameter Estimates

		Estimate	Std. Error	Wald	df	Sig.	95% Confidence Interval	
							Lower Bound	Upper Bound
Threshold	[Liveonline = 1.00]	-3.226	.209	237.612	1	<.001	-3.636	-2.816
	[Liveonline = 2.00]	-1.440	.162	79.451	1	<.001	-1.757	-1.123
	[Liveonline = 3.00]	-.260	.154	2.839	1	.092	-.563	.042
Location	[UG_or_PG=1.00]	-.241	.167	2.079	1	.149	-.569	.087
	[UG_or_PG=2.00]	0[a]	.	.	0	.	.	.

Link function: Logit.

a. This parameter is set to zero because it is redundant.

Cell Information

Frequency

UG_or_PG		Liveonline 1.00	2.00	3.00	4.00
Under-graduate level	Observed	37	149	216	407
	Expected	38.908	148.481	213.227	408.384
	Pearson Residual	-.314	.047	.221	-.097
Post-graduate level	Observed	8	24	36	91
	Expected	6.072	24.382	38.763	89.784
	Pearson Residual	.798	-.084	-.510	.195

Link function: Logit.

Test of Parallel Lines[a]

Model	-2 Log Likelihood	Chi-Square	df	Sig.
Null Hypothesis	33.571			
General	32.653	.918	2	.632

The null hypothesis states that the location parameters (slope coefficients) are the same across response categories.

a. Link function: Logit.

PLUM - Ordinal Regression

Case Processing Summary

		N	Marginal Percentage
PreRecordedLecturesF2Ftutorials	1.00	319	33.0%
	2.00	421	43.5%
	3.00	160	16.5%
	4.00	68	7.0%
UG_or_PG	Under-graduate level	809	83.6%
	Post-graduate level	159	16.4%
Valid		968	100.0%
Missing		169	
Total		1137	

Model Fitting Information

Model	-2 Log Likelihood	Chi-Square	df	Sig.
Intercept Only	38.220			
Final	37.245	.975	1	.324

Link function: Logit.

Goodness-of-Fit

	Chi-Square	df	Sig.
Pearson	3.822	2	.148
Deviance	3.757	2	.153

Link function: Logit.

Pseudo R-Square

Cox and Snell	.001
Nagelkerke	.001
McFadden	.000

Link function: Logit.

Parameter Estimates

		Estimate	Std. Error	Wald	df	Sig.	95% Confidence Interval Lower Bound	95% Confidence Interval Upper Bound
Threshold	[PreRecordedLecturesF2Ftutorials = 1.00]	-.578	.150	14.796	1	<.001	-.872	-.283
	[PreRecordedLecturesF2Ftutorials = 2.00]	1.312	.156	71.105	1	<.001	1.007	1.617
	[PreRecordedLecturesF2Ftutorials = 3.00]	2.718	.186	214.265	1	<.001	2.354	3.082
Location	[UG_or_PG=1.00]	.159	.161	.980	1	.322	-.156	.475
	[UG_or_PG=2.00]	0[a]	.	.	0	.	.	.

Link function: Logit.

a. This parameter is set to zero because it is redundant.

Cell Information

Frequency

UG_or_PG		PreRecordedLecturesF2Ftutorials 1.00	2.00	3.00	4.00
Under-graduate level	Observed	262	351	142	54
	Expected	261.838	352.999	136.051	58.112
	Pearson Residual	.012	-.142	.559	-.560
Post-graduate level	Observed	57	70	18	14
	Expected	57.154	68.113	23.890	9.844
	Pearson Residual	-.025	.302	-1.307	1.368

Link function: Logit.

Test of Parallel Lines[a]

Model	-2 Log Likelihood	Chi-Square	df	Sig.
Null Hypothesis	37.245			
General	33.488	3.757	2	.153

The null hypothesis states that the location parameters (slope coefficients) are the same across response categories.

a. Link function: Logit.

PLUM - Ordinal Regression

Case Processing Summary

		N	Marginal Percentage
PreRecordedLecturesLivetutorials	1.00	96	9.9%
	2.00	165	17.0%
	3.00	430	44.4%
	4.00	277	28.6%
UG_or_PG	Under-graduate level	809	83.6%
	Post-graduate level	159	16.4%
Valid		968	100.0%
Missing		169	
Total		1137	

Model Fitting Information

Model	-2 Log Likelihood	Chi-Square	df	Sig.
Intercept Only	45.544			
Final	34.377	11.167	1	<.001

Link function: Logit.

Goodness-of-Fit

	Chi-Square	df	Sig.
Pearson	.187	2	.911
Deviance	.188	2	.910

Link function: Logit.

Pseudo R-Square

Cox and Snell	.011
Nagelkerke	.012
McFadden	.005

Link function: Logit.

Parameter Estimates

		Estimate	Std. Error	Wald	df	Sig.	95% Confidence Interval	
							Lower Bound	Upper Bound
Threshold	[PreRecordedLecturesLivetutorials = 1.00]	-1.779	.166	115.042	1	<.001	-2.104	-1.454
	[PreRecordedLecturesLivetutorials = 2.00]	-.562	.149	14.297	1	<.001	-.853	-.271
	[PreRecordedLecturesLivetutorials = 3.00]	1.367	.155	78.147	1	<.001	1.064	1.669
Location	[UG_or_PG=1.00]	.532	.160	11.074	1	<.001	.219	.845
	[UG_or_PG=2.00]	0[a]	.	.	0	.	.	.

Link function: Logit.

a. This parameter is set to zero because it is redundant.

Test of Parallel Lines[a]

Model	-2 Log Likelihood	Chi-Square	df	Sig.
Null Hypothesis	34.377			
General	34.189	.188	2	.910

The null hypothesis states that the location parameters (slope coefficients) are the same across response categories.

a. Link function: Logit.

Generalized Linear Models

Model Information

Dependent Variable	PreRecordedLecturesLivetutorials[a]
Probability Distribution	Multinomial
Link Function	Cumulative logit

a. The procedure applies the cumulative link function to the dependent variable values in ascending order.

Case Processing Summary

	N	Percent
Included	968	85.1%
Excluded	169	14.9%
Total	1137	100.0%

Categorical Variable Information

			N	Percent
Dependent Variable	PreRecordedLecturesLivetutorials	1.00	96	9.9%
		2.00	165	17.0%
		3.00	430	44.4%
		4.00	277	28.6%
		Total	968	100.0%
Factor	UG_or_PG	Under-graduate level	809	83.6%
		Post-graduate level	159	16.4%
		Total	968	100.0%

Goodness of Fit[a]

	Value	df	Value/df
Deviance	.188	2	.094
Scaled Deviance	.188	2	
Pearson Chi-Square	.187	2	.094
Scaled Pearson Chi-Square	.187	2	
Log Likelihood[b]	-17.189		
Akaike's Information Criterion (AIC)	42.377		
Finite Sample Corrected AIC (AICC)	42.419		
Bayesian Information Criterion (BIC)	61.878		

Consistent AIC (CAIC)	65.878		

Dependent Variable: PreRecordedLecturesLivetutorials

Model: (Threshold), UG_or_PG[a]

a. Information criteria are in smaller-is-better form.

b. The full log likelihood function is displayed and used in computing information criteria.

Omnibus Test[a]

Likelihood Ratio Chi-Square	df	Sig.
11.167	1	<.001

Dependent Variable: PreRecordedLecturesLivetutorials

Model: (Threshold), UG_or_PG[a]

a. Compares the fitted model against the thresholds-only model.

Tests of Model Effects

Source	Type III Likelihood Ratio Chi-Square	df	Sig.
UG_or_PG	11.167[a]	1	<.001

Dependent Variable: PreRecordedLecturesLivetutorials

Model: (Threshold), UG_or_PG

a. The validity of the likelihood ratio chi-square is uncertain because log-likelihood convergence was not achieved for the constrained model. Results shown are based on the last iteration.

Parameter Estimates

Parameter		B	Std. Error	95% Wald Confidence Interval Lower	95% Wald Confidence Interval Upper	Hypothesis Test Wald Chi-Square	Hypothesis Test df	Hypothesis Test Sig.	Exp(B)	95% Wald Confidence Interval for Exp(B) Lower	95% Wald Confidence Interval for Exp(B) Upper
Threshold	[PreRecordedLecturesLivetutorials=1.00]	-1.779	.1653	-2.103	-1.455	115.895	1	.000	.169	.122	.233
	[PreRecordedLecturesLivetutorials=2.00]	-.562	.1477	-.851	-.272	14.467	1	<.001	.570	.427	.762
	[PreRecordedLecturesLivetutorials=3.00]	1.367	.1538	1.065	1.668	78.923	1	.000	3.922	2.901	5.302
[UG_or_PG=1.00]		.532	.1591	.220	.844	11.175	1	<.001	1.702	1.246	2.325
[UG_or_PG=2.00]		0[a]	.	.	.	.	.	.	1	.	.
(Scale)		1[b]									

Dependent Variable: PreRecordedLecturesLivetutorials

Model: (Threshold), UG_or_PG

a. Set to zero because this parameter is redundant.

b. Fixed at the displayed value.

Data for ordinal regression considering biological sex.

PLUM - Ordinal Regression

Case Processing Summary

		N	Marginal Percentage
F2F_Lec_Tuto	1.00	357	50.7%
	2.00	156	22.2%
	3.00	99	14.1%
	4.00	92	13.1%
Gender_groups	male	207	29.4%
	female	497	70.6%
Valid		704	100.0%
Missing		69	
Total		773	

Model Fitting Information

Model	-2 Log Likelihood	Chi-Square	df	Sig.
Intercept Only	38.089			
Final	35.653	2.436	1	.119

Link function: Logit.

Goodness-of-Fit

	Chi-Square	df	Sig.
Pearson	2.471	2	.291
Deviance	2.491	2	.288

Link function: Logit.

Pseudo R-Square

Cox and Snell	.003
Nagelkerke	.004
McFadden	.001

Link function: Logit.

Parameter Estimates

		Estimate	Std. Error	Wald	df	Sig.	95% Confidence Interval Lower Bound	95% Confidence Interval Upper Bound
Threshold	[F2F_Lec_Tuto = 1.00]	-.042	.088	.223	1	.637	-.214	.131
	[F2F_Lec_Tuto = 2.00]	.921	.095	93.492	1	<.001	.734	1.108
	[F2F_Lec_Tuto = 3.00]	1.829	.120	234.029	1	<.001	1.594	2.063
Location	[Gender_groups=1.00]	-.246	.157	2.461	1	.117	-.554	.061
	[Gender_groups=2.00]	0[a]	.	.	0	.	.	.

Link function: Logit.

a. This parameter is set to zero because it is redundant.

Cell Information

Frequency

Gender_groups		F2F_Lec_Tuto 1.00	2.00	3.00	4.00
male	Observed	117	37	26	27
	Expected	114.067	43.815	26.028	23.091
	Pearson Residual	.410	-1.160	-.006	.863
female	Observed	240	119	73	65
	Expected	243.335	112.155	72.719	68.791
	Pearson Residual	-.299	.735	.036	-.492

Link function: Logit.

Test of Parallel Lines[a]

Model	-2 Log Likelihood	Chi-Square	df	Sig.
Null Hypothesis	35.653			
General	33.162	2.491	2	.288

The null hypothesis states that the location parameters (slope coefficients) are the same across response categories.

a. Link function: Logit.

PLUM - Ordinal Regression

Case Processing Summary

		N	Marginal Percentage
Liveonline	1.00	38	5.4%
	2.00	126	17.9%
	3.00	179	25.4%
	4.00	361	51.3%
Gender_groups	male	207	29.4%
	female	497	70.6%
Valid		704	100.0%
Missing		69	
Total		773	

Model Fitting Information

Model	-2 Log Likelihood	Chi-Square	df	Sig.
Intercept Only	32.701			
Final	32.339	.362	1	.547

Link function: Logit.

Goodness-of-Fit

	Chi-Square	df	Sig.
Pearson	.090	2	.956
Deviance	.090	2	.956

Link function: Logit.

Pseudo R-Square

Cox and Snell	.001
Nagelkerke	.001
McFadden	.000

Link function: Logit.

Parameter Estimates

		Estimate	Std. Error	Wald	df	Sig.	95% Confidence Interval	
							Lower Bound	Upper Bound
Threshold	[Liveonline = 1.00]	-2.837	.173	270.370	1	<.001	-3.175	-2.499
	[Liveonline = 2.00]	-1.164	.100	136.127	1	<.001	-1.360	-.969
	[Liveonline = 3.00]	-.023	.088	.070	1	.791	-.196	.150
Location	[Gender_groups=1.00]	.094	.157	.360	1	.549	-.213	.401
	[Gender_groups=2.00]	0[a]	.	.	0	.	.	.

Link function: Logit.

a. This parameter is set to zero because it is redundant.

Cell Information

Frequency

Gender_groups		Liveonline 1.00	2.00	3.00	4.00
male	Observed	10	35	53	109
	Expected	10.485	35.314	51.633	109.569
	Pearson Residual	-.154	-.058	.220	-.079
female	Observed	28	91	126	252
	Expected	27.518	90.707	127.372	251.403
	Pearson Residual	.095	.034	-.141	.054

Link function: Logit.

Test of Parallel Lines[a]

Model	-2 Log Likelihood	Chi-Square	df	Sig.
Null Hypothesis	32.339			
General	32.249	.090	2	.956

The null hypothesis states that the location parameters (slope coefficients) are the same across response categories.

a. Link function: Logit.

PLUM - Ordinal Regression

Case Processing Summary

		N	Marginal Percentage
PreRecordedLecturesLivetutorials	1.00	62	8.8%
	2.00	127	18.0%
	3.00	313	44.5%
	4.00	202	28.7%
Gender_groups	male	207	29.4%
	female	497	70.6%
Valid		704	100.0%
Missing		69	
Total		773	

Model Fitting Information

Model	-2 Log Likelihood	Chi-Square	df	Sig.
Intercept Only	35.043			
Final	34.915	.128	1	.720

Link function: Logit.

Goodness-of-Fit

	Chi-Square	df	Sig.
Pearson	1.714	2	.424
Deviance	1.728	2	.421

Link function: Logit.

Pseudo R-Square

Cox and Snell	.000
Nagelkerke	.000
McFadden	.000

Link function: Logit.

Parameter Estimates

		Estimate	Std. Error	Wald	df	Sig.	95% Confidence Interval Lower Bound	Upper Bound
Threshold	[PreRecordedLecturesLivetutorials = 1.00]	-2.354	.141	279.913	1	<.001	-2.630	-2.078
	[PreRecordedLecturesLivetutorials = 2.00]	-1.019	.096	111.645	1	<.001	-1.208	-.830
	[PreRecordedLecturesLivetutorials = 3.00]	.894	.094	89.756	1	<.001	.709	1.079
Location	[Gender_groups=1.00]	-.054	.153	.126	1	.722	-.353	.245
	[Gender_groups=2.00]	0[a]	.	.	0	.	.	.

Link function: Logit.

a. This parameter is set to zero because it is redundant.

Cell Information

Frequency

		PreRecordedLecturesLivetutorials			
Gender_groups		1.00	2.00	3.00	4.00
male	Observed	16	38	99	54
	Expected	18.868	38.246	92.087	57.800
	Pearson Residual	-.692	-.044	.967	-.589
female	Observed	46	89	214	148
	Expected	43.119	88.695	220.928	144.258
	Pearson Residual	.459	.036	-.625	.370

Link function: Logit.

Test of Parallel Lines[a]

Model	-2 Log Likelihood	Chi-Square	df	Sig.
Null Hypothesis	34.915			
General	33.187	1.728	2	.421

The null hypothesis states that the location parameters (slope coefficients) are the same across response categories.

a. Link function: Logit.

PLUM - Ordinal Regression

Case Processing Summary

		N	Marginal Percentage
PreRecordedLecturesF2Ftutorials	1.00	245	34.8%
	2.00	291	41.3%
	3.00	118	16.8%
	4.00	50	7.1%
Gender_groups	male	207	29.4%
	female	497	70.6%
Valid		704	100.0%
Missing		69	
Total		773	

Model Fitting Information

Model	-2 Log Likelihood	Chi-Square	df	Sig.
Intercept Only	38.179			
Final	37.674	.505	1	.477

Link function: Logit.

Goodness-of-Fit

	Chi-Square	df	Sig.
Pearson	4.718	2	.095
Deviance	4.775	2	.092

Link function: Logit.

Pseudo R-Square

Cox and Snell	.001
Nagelkerke	.001
McFadden	.000

Link function: Logit.

Parameter Estimates

		Estimate	Std. Error	Wald	df	Sig.	95% Confidence Interval Lower Bound	95% Confidence Interval Upper Bound
Threshold	[PreRecordedLecturesF2Ftutorials = 1.00]	-.596	.091	43.207	1	<.001	-.773	-.418
	[PreRecordedLecturesF2Ftutorials = 2.00]	1.193	.100	143.008	1	<.001	.998	1.389
	[PreRecordedLecturesF2Ftutorials = 3.00]	2.604	.154	285.638	1	<.001	2.302	2.905
Location	[Gender_groups=1.00]	.108	.152	.498	1	.480	-.191	.406
	[Gender_groups=2.00]	0^a	.	.	0	.	.	.

Link function: Logit.

a. This parameter is set to zero because it is redundant.

Cell Information

Frequency

Gender_groups		PreRecordedLecturesF2Ftutorials 1.00	2.00	3.00	4.00
male	Observed	64	97	29	17
	Expected	68.545	86.195	36.499	15.761
	Pearson Residual	-.671	1.523	-1.368	.325
female	Observed	181	194	89	33
	Expected	176.620	204.723	81.409	34.249
	Pearson Residual	.411	-.977	.920	-.221

Link function: Logit.

Test of Parallel Lines[a]

Model	-2 Log Likelihood	Chi-Square	df	Sig.
Null Hypothesis	37.674			
General	32.899	4.775	2	.092

The null hypothesis states that the location parameters (slope coefficients) are the same across response categories.

a. Link function: Logit.
